# Chemical Design and Magnetic Ordering in Thin Layers of 2D Metal–Organic Frameworks (MOFs)

**DOI:** 10.1021/jacs.1c07802

**Published:** 2021-11-01

**Authors:** Javier López-Cabrelles, Samuel Mañas-Valero, Iñigo J. Vitórica-Yrezábal, Makars Šiškins, Martin Lee, Peter G. Steeneken, Herre S. J. van der Zant, Guillermo Mínguez Espallargas, Eugenio Coronado

**Affiliations:** † Instituto de Ciencia Molecular (ICMol), Universidad de Valencia, c/Catedrático José Beltrán, 2, 46980 Paterna, Spain; ‡ School of Chemistry, University of Manchester, Oxford Road, Manchester M13 9PL, U.K.; § Kavli Institute of Nanoscience, Delft University of Technology, Lorentzweg 1, 2628 CJ, Delft, The Netherlands; ∥ Department of Precision and Microsystems Engineering, Delft University of Technology, Mekelweg 2, 2628 CD, Delft, The Netherlands

## Abstract

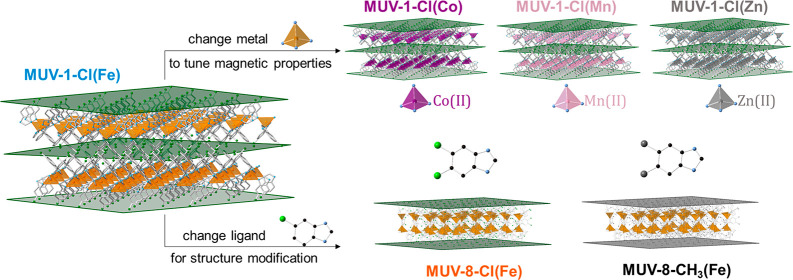

Through rational chemical design, and thanks to the hybrid nature of metal–organic frameworks (MOFs), it is possible to prepare molecule-based 2D magnetic materials stable at ambient conditions. Here, we illustrate the versatility of this approach by changing both the metallic nodes and the ligands in a family of layered MOFs that allows the tuning of their magnetic properties. Specifically, the reaction of benzimidazole-type ligands with different metal centers (M^II^ = Fe, Co, Mn, Zn) in a solvent-free synthesis produces a family of crystalline materials, denoted as MUV-1(M), which order antiferromagnetically with critical temperatures that depend on M. Furthermore, the incorporation of additional substituents in the ligand results in a novel system, denoted as MUV-8, formed by covalently bound magnetic double layers interconnected by van der Waals interactions, a topology that is very rare in the field of 2D materials and unprecedented for 2D magnets. These layered materials are robust enough to be mechanically exfoliated down to a few layers with large lateral dimensions. Finally, the robustness and crystallinity of these layered MOFs allow the fabrication of nanomechanical resonators that can be used to detect—through laser interferometry—the magnetic order in thin layers of these 2D molecule-based antiferromagnets.

## Introduction

The isolation of atomically thin—single or few-layer—crystals has given rise to the emergence of the so-called two-dimensional (2D) materials. These low-dimensional materials have shown a wide range of electronic and magnetic properties (from insulators to superconductors; from ferromagnets to antiferromagnets and quantum spin liquids) that can be affected by the dimensionality.^1−5^ Most of these materials derive from well-known layered inorganic materials,^6^ formed by covalently bound layers interconnected by weak van der Waals interactions, allowing the isolation of monolayers by exfoliation. A hot topic in this area deals with the isolation of monolayers of magnets with the aim of studying magnetism in the 2D limit and to integrate these layers in spintronic devices.^7,8^ The exploration of such experimental possibilities is hindered by the chemical instability in open air of the chosen inorganic compounds (mainly based on layered metal halides), thus limiting their manipulation and applicability. In fact, the first report on the magnetism of CrI_3_ down to the monolayer limit was published in 2017^9^ even though the discovery of its bulk properties has been known since 1959.^10^

Coordination chemistry can also offer a source of layered metal–organic materials which, in contrast to the inorganic analogs, are much more versatile from the point of view of the chemical design, chemical stability, and ease of manipulation in open air. However, the isolation of monolayers of these molecule-based magnets has been achieved in very few cases only.^11^ This may be due to the hybrid nature of the materials, which in most cases are formed by charged layers interleaved by counterions. Hence, these layers are held together by electrostatic interactions and not by van der Waals interactions. This often results in fragile crystals of small sizes, which are very difficult to exfoliate using a micromechanical Scotch tape procedure. In fact, the first successful mechanical exfoliation of a layered magnet of this kind was reported by us in 2015 in the coordination polymer [Fe^III^(acac_2_-trien)][Mn^II^Cr^III^(anilate)_3_]·(CH_3_CN)_2_, where anilate refers to dichloro- and dibromo-substituted anilate ligand.^12^ These compounds in bulk behave as ferrimagnets with a transition temperature, *T*_c_ = 11 K. The structure of the magnetic layers consists of a hexagonal 2D anionic network formed by Mn^2+^ and Cr^3+^ ions linked through anilate ligands. In this case, and thanks to the large size of the hexagonal pores, the counterions [Fe^III^(acac_2_-trien)]^+^ were inserted inside the magnetic framework instead of being in the interlamellar space. This feature facilitated the micromechanical exfoliation, leading to the isolation of magnetic layers with thicknesses down to 1.5 nm and lateral sizes on the order of hundreds of nanometers.

More recently, this result has been drastically improved by designing coordination compounds formed by neutral layers.^13^ To reach this goal we have exploited the chemical versatility provided by imidazolate-type ligands in the design of metal organic frameworks (MOFs) of various dimensionalities and properties.^14−16^ Thus, a isoreticular series of layered magnetic MOFs, composed by benzimidazole derivates and Fe(II) centers (**MUV-1-X(Fe)**, with X = H, Cl, Br, CH_3_ or NH_2_) and behaving as spin-canted antiferromagnets with ordering Neel temperatures, *T*_N_, of ∼20 K, has been synthesized by a solvent-free method.^13^ In this series, monolayers have been isolated through micromechanical exfoliation, reaching high-quality crystalline flakes of micron size. On the other hand, we have also shown that these materials can be exfoliated in large amounts by using a liquid exfoliation method.^17^

**Figure 1:**
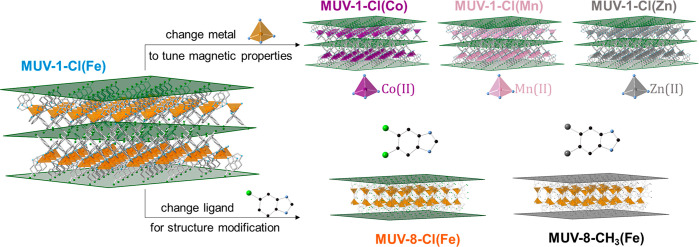
Chemical versatility of the MUV-1 system: it is possible to modify the magnetic properties by changing the metallic cation (upper part) and to induce structural changes by adding a second substituent to the benzimidazole ligand (bottom part), while keeping the layered morphology needed for a two-dimensional material.

Interestingly, the chemical versatility of this molecular approach has allowed us to functionalize the magnetic layer at will by changing the substituent X in the benzimidazole, while keeping its magnetic properties unchanged.^13^ Herein, we further exploit this versatility either by changing the metallic nodes, while maintaining the crystal structure, or by inserting a second substituent in the ligand, while retaining the layered morphology of the material (Figure 1). The first possibility provides the opportunity to tune the magnetic properties of the layers, while the second results in the isolation of covalently bound magnetic double-layers interconnected by van der Waals interactions, a topology that is very rare in the field of 2D materials and unprecedented for 2D magnets. In the second part of this work, these van der Waals antiferromagnets are mechanically exfoliated down to the atomically thin layers and we show that their magnetic ordering can be probed mechanically by nanomechanical resonators made of thin membranes of these insulating materials. Compared to ferromagnets, antiferromagnets exhibit high frequency magnon dynamics and are insensitive to external magnetic fields. Since most of them are insulators, they can present pure spin currents with no Joule heating, making them of potential interest for low-consumption spintronic memory devices.^18^ In the particular case of 2D antiferromagnets a larger variety of spin anisotropies, which can be optically controlled, have been experimentally shown.^19^

## Results and Discussion

### Changing Metallic Nodes

**Figure 2:**
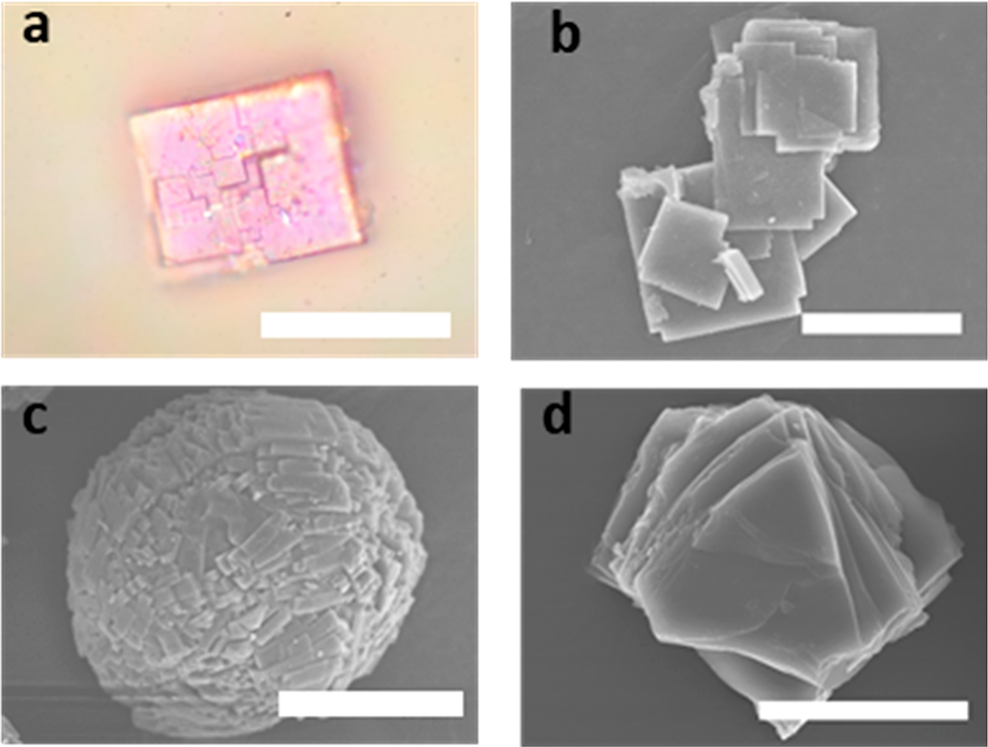
(a) Optical images for a crystal of **MUV-1-Cl(Co**). (b,c) SEM images of **MUV-1-Cl(Co)** and (d) **MUV-8-Cl(Fe)** showing the layered morphology. Note that **MUV-1-Cl(Co)** presents spontaneous crystal aggregation with spherical uncommon morphologies. Scale bars are 10, 10, 20, and 30 μm, respectively.

The existence of different metal sources compatible with chemical vapor deposition and solvent-free methods permits the modification of the reaction previously described in ref (13), where benzimidazole derivates (HbimX, X = Cl, Br, CH_3_, H, NH_2_) and ferrocene were employed, using different metal cyclopentadienyls and other metal precursors (see details in Section S1). Obtaining isostructural materials changing the metal node is a challenge that leads to the modification of the properties of the materials, in the present case, the magnetic properties (Figure 1). Biscyclopentadienyl cobalt(II), bis(tetramethylcyclopentadienyl) manganese(II), bis(2,2,6,6-tetramethyl-3,5-heptanedionato) zinc(II), and ZnO were used as metal precursors in a solvent-free method to synthesize isostructural compounds of **MUV-1-Cl** and **MUV-1-H**. The crystals obtained in the synthesis were characterized by single crystal X-ray diffraction and/or powder X-ray diffraction (see Sections S2 and S3), obtaining three isostructural compounds of **MUV-1-Cl** (Co, Mn, and Zn) and three of **MUV-1-H** (Co, Mn, and Zn). The metal cation is located in the inner part of the layers in a distorted tetrahedral environment, connected by benzimidazole bridges, allowing magnetic exchange between the metal centers. The size of the crystals is smaller for the cobalt, manganese, and zinc compounds than for the previously reported iron analogue, but all of these materials keep the layered morphology (Figures 2 and S1–S3). These changes in the metallic nodes introduce changes in both the single-ion magnetic anisotropy and the intralayer exchange interactions that permit the tunability of the magnetic properties in these materials (see Magnetic Properties section).

### Changing the Ligands

**Figure 3:**
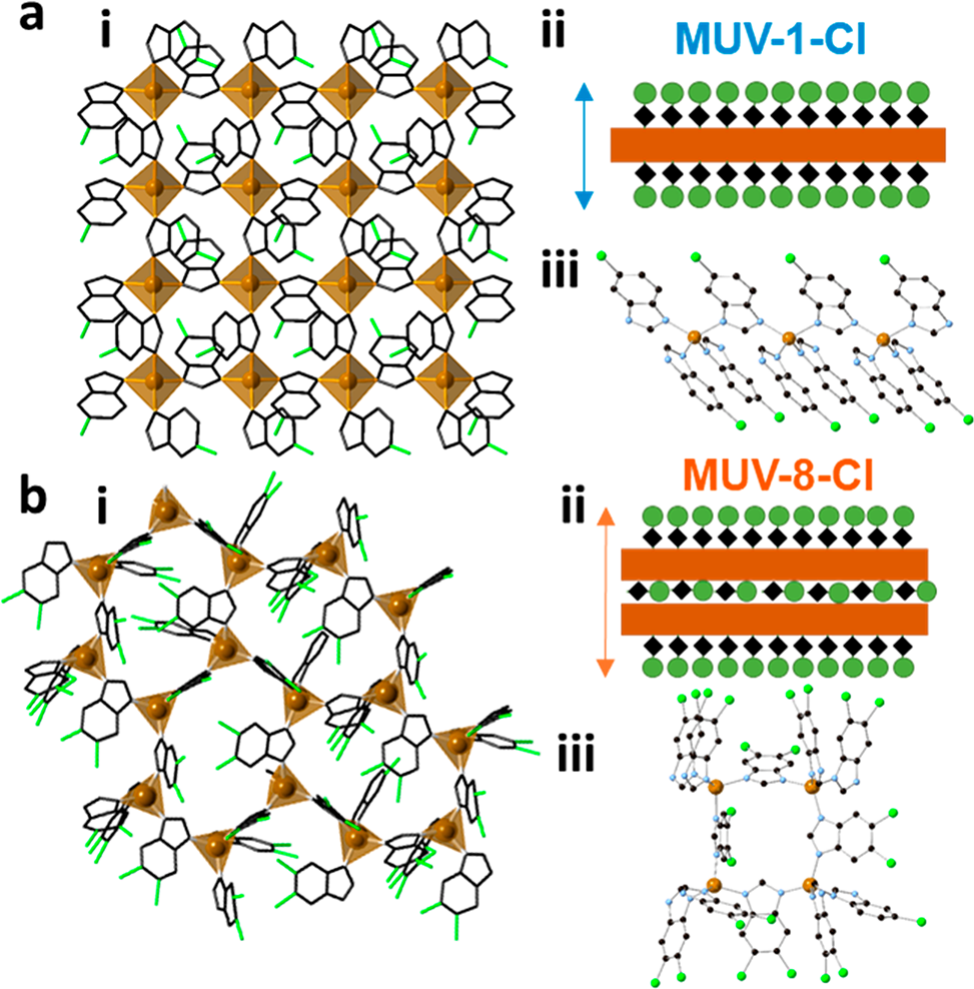
Comparison of the crystal structures of **MUV-1-Cl** (a) and **MUV-8-Cl** (b). Structure of a single layer (**i**) viewed along the *c* axis (*ab* plane). Schematic representation of a single layer (**ii**), showing an increase thickness for the “double layer” of irons in the **MUV-8-Cl** (from 1 to 1.5 nm). Coordination modes of the ligands (**iii**).

The molecular nature of the coordination polymers permits changes not only in the metal nodes but also in the ligand part (i.e., on the functional groups). In this case, a second substituent is added in the organic ligand adjacent to the 5 position where the initial substituent is present. Thus, two different ligands have been used, 5,6-dichlorobenzimidazole (HbimCl_2_) and 5,6-dimethylbenzimidazole (Hbim(CH_3_)_2_). Importantly, this change in the ligand does not affect the formation of a layered structure, and their reaction with ferrocene gives rise to layered coordination polymers, the so-called **MUV-8-Cl(Fe)** and **MUV-8-CH**_**3**_**(Fe)** (Figures 2 and S1). However, this second functional group induces a significant structural change resulting in unprecedented double layers of iron(II) centers arranged in distorted hexagons and linked through bridging benzimidazole ligands (Figure 3).

There are four crystallographically different iron centers in a single double layer of **MUV-8** (Figure S2). These iron centers are connected by three bidentate ligands in the *ab* plane, and a fourth bidentate ligand connecting two iron centers each in its respective layer (in the *c* direction) (Figure 3biii). As a consequence, the resulting double layer in **MUV-8** exhibits a thickness of 1.5 nm, being formed by the sequence “ligand layer/Fe layer/ligand layer/Fe layer/ligand layer”, which is large compared to the thickness of 1 nm in **MUV-1** since that is formed just by one monolayer of Fe centers (sequence of “ligand layer/Fe layer/ligand layer”). The iron centers are located in very distorted tetrahedral sites, with Fe–N lengths of 1.8–2.15 Å, and connected by benzimidazolate bridges.

These connected layers are superposed in a similar arrangement to an AB stacking, with a small shift between iron centers (Figure S2). The chlorine atoms belonging to the ligands placed in the *ab* plane are pointing toward the surfaces and are weakly interacting through van der Waals forces like in the **MUV-1-Cl** case, while the bridging ligand connecting the two monolayers is located inside of the double layer. Moreover, in **MUV-8-X**, some ferrocene molecules are located between the layers, interacting weakly with the layers of the compound (Figure S3). These molecules cannot be removed with heat as can be seen by thermogravimetric analysis (Figure S6). The two-dimensional units are composed of covalently bound double layers held together through coordination bonds and interconnected by weak van der Waals interactions, instead of being formed by covalently bound monolayers. This novel structural arrangement opens the way to isolate unprecedented 2D magnetic networks using a coordination chemistry approach.

**Figure 4:**
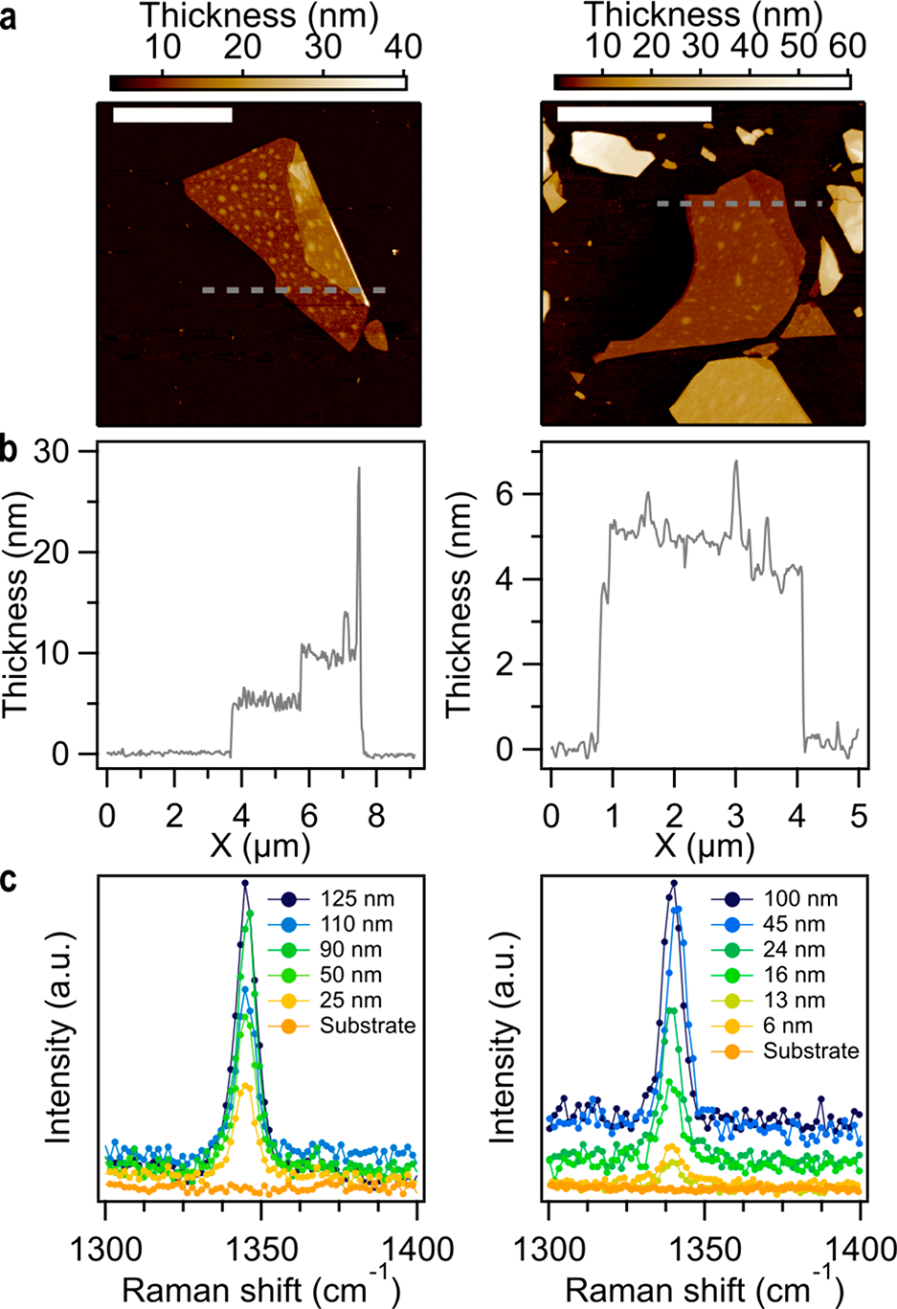
(a,b) AFM image and profile of a typical exfoliated **MUV-1-Cl(Co)** (left) and **MUV-8-Cl(Fe)** flake (right). Scale bars are 5 μm. (c) Selected region of the Raman spectra of **MUV-1-Cl(Co)** (left) and **MUV-8-Cl(Fe)** (right) flakes of different thicknesses.

The layered morphology of the crystals (Figure 2) allows their delamination and the possibility to explore the 2D limit in these compounds. We have focused on the cobalt system, **MUV-1-Cl(Co)** and **MUV-1-H(Co)**, and on **MUV-8-Cl(Fe)** and **MUV-8-CH**_**3**_**(Fe)**. Bulk crystals of these four systems were thinned-down by mechanical exfoliation as previously realized in **MUV-1-Cl**,^13^ yielding flakes with well-defined shapes (lateral dimensions of >1 μm) and different thicknesses (from hundreds of nanometers down to a few nanometers). The obtained flakes are characterized by optical and atomic force (AFM) microscopies (Figure 4a and 4b, and Section S5) as well as Raman spectroscopy, in order to confirm their integrity and chemical composition. In the case of the novel structures of **MUV-8**, we have achieved thin-layer with a thickness of 6 nm, which in this case corresponds to 3–4 monolayers. Importantly, we have been able to detect the Raman spectra of these ultrathin films (Figure 4c).

### Magnetic Properties

**Figure 5:**
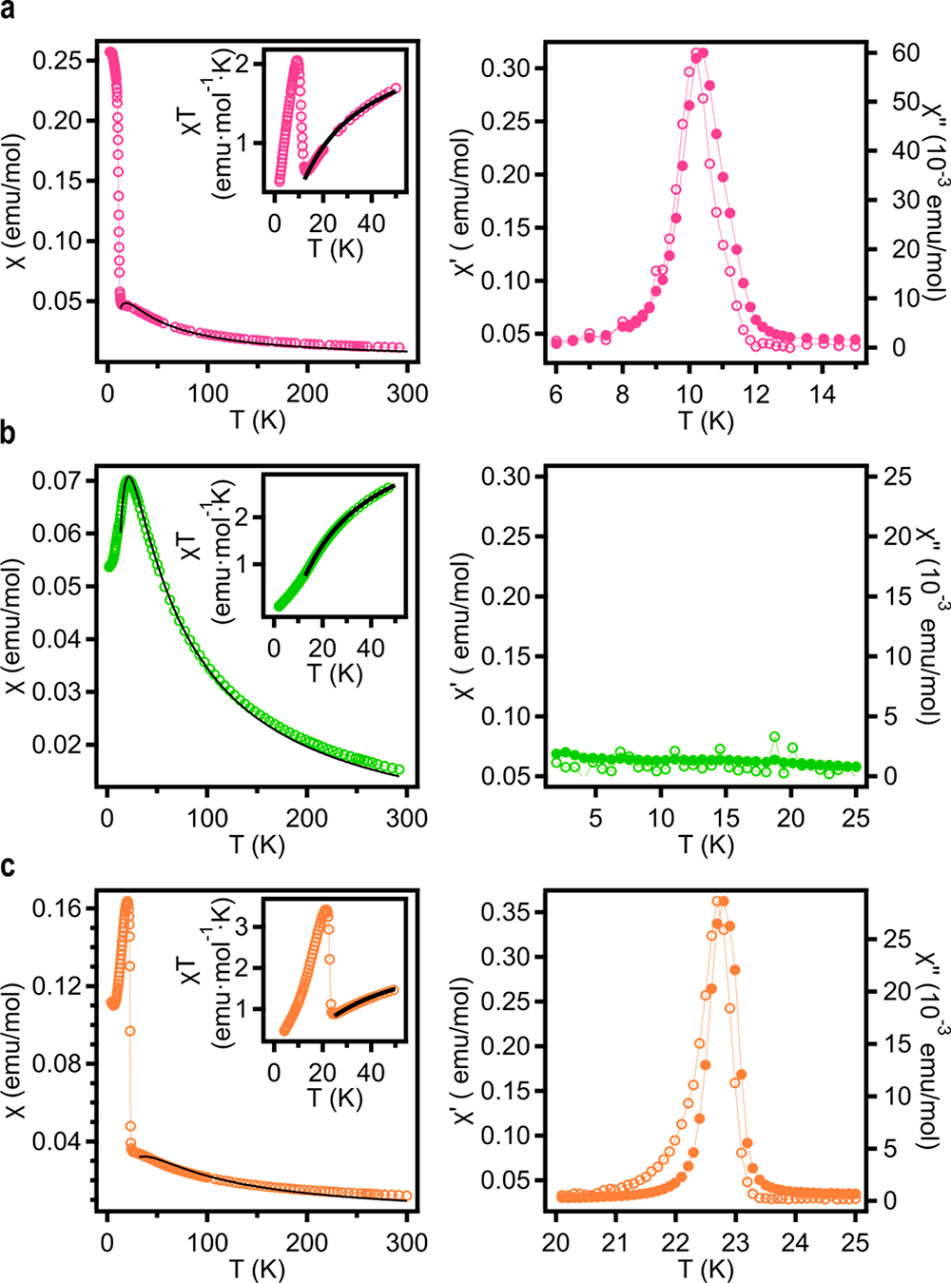
d.c. (left column) and a.c. (right column; in-phase and out-of-phase components at 110 Hz are denoted with solid and open circles, respectively) magnetic susceptibilities for different **MUV-1** and **MUV-8** compounds. (a) **MUV-1-Cl(Co)**, (b) **MUV-1-H(Mn)**, (c) **MUV-8-Cl(Fe)**. Solid black lines indicate the fit in the low dimensional regime to the expression for an antiferromagnetic square lattice (**MUV-1**) and the Curély formula for an antiferromagnetic hexagonal layer (**MUV-8**).^21,22^

The magnetic behavior of the polycrystalline bulk samples was investigated by SQUID measurements in the range 2–300 K. Respective data can be found in the Supporting Information (SI) (see Section S4). In Figure 5 we plot the thermal variation of the susceptibility (χ) for the Co(II) and Mn(II) derivatives of **MUV-1**, as well as the Fe(II) derivative of **MUV-8**. In these compounds χ increases upon cooling down until a maximum or a levelling are reached at intermediate temperatures. This agrees with an antiferromagnetic coupling between the metal centers, also supported by the continuous decrease in the χ*T* product in this region (insets of the Figure). At lower temperatures, a sharp increase in χ is observed for the Co and Fe samples. This feature, together with the presence of a sharp out-of-phase component of the a.c. susceptibility signal, χ′′, at this temperature (Figure 5, right) and magnetic hysteresis loops (Figure S18), is indicative of a transition toward antiferromagnetic order with spin canting at the critical temperature, *T*_N_. In contrast, in the Mn derivative, the maximum in χ is followed by a sharp decrease at lower temperature, which agrees with an antiferromagnetic ordered structure without canting (Figure 5, left). This is corroborated by the absence of a χ′′ signal in the vicinity of *T*_N_ (Figure 5, right). Such a difference may be related to the fully isotropic nature of the spin in the Mn derivative (with a ground term ^6^A_1_), while some magnetic anisotropy is expected for high-spin Co(II) and Fe(II) in tetrahedral sites (described by ^4^A_2_ and ^5^E terms, respectively).

The critical temperatures in these antiferromagnets have been estimated from the temperature where χ″ differs from 0 in the case of systems with spin-canting (**MUV-1(Co)** and **MUV-8(Fe)**). In the case where the canting is absent (**MUV-1(Mn)**), *T*_N_ is estimated, according to the Fisher criteria,^20^ by considering the maximum in ∂(χ*T*)/∂*T*. In this last system, a *T*_N_ value of 14 K is derived, which is consistent with the EPR measurements that show important changes in the signal near this temperature (see Section S4.2). *T*_N_ values for all the materials are summarized in Table 1.

**Table 1:** Magnetic Properties of the **MUV-1** and **MUV-8** Families^a^

MUV	*S*	*T*_N_ (K)	*J* (cm^–1^)	*g*	*C* (emu·mol^–1^·K)	Θ (K)
MUV-1-Cl(Fe)^b^	2	20.7	–22.9 ± 0.4	2.00 ± 0.02	3.635 ± 0.006	–80.6 ± 0.4
MUV-1-H(Fe)^b^	2	20.0	–23.5 ± 0.2	1.98 ± 0.02	5.76 ± 0.02	–97.5 ± 1.3
MUV-1-Br(Fe)^b^	2	20.0	–22.8 ± 0.2	2.0 ± 0.2	3.693 ± 0.002	–89.32 ± 0.2
MUV-1-CH_3_(Fe)^b^	2	20.1	–22.6 ± 0.4	2.0 ± 0.2	5.13 ± 0.03	–132.0 ± 1.9
MUV-1-NH_2_(Fe)^b^	2	21.2	–23.3 ± 0.3	2.0 ± 0.2	3.976 ± 0.014	–106.8 ± 1.5
MUV-1-Cl(Co)	3/2	11.6	–20.2 ± 0.4	2.3 ± 0.2	3.915 ± 0.044	–73 ± 2
MUV-1-H(Co)	3/2	12.4	–20.8 ± 0.4	2.2 ± 0.2	4.11 ± 0.07	–112 ± 4
MUV-1-Cl(Mn)	5/2	14.3	–10.7 ± 0.2	2.2 ± 0.2	6.60 ± 0.05	–65.9 ± 1.5
MUV-1-H(Mn)	5/2	14.8	–10.4 ± 0.2	2.02 ± 0.10	5.138 ± 0.012	–46.7 ± 0.4
MUV-8-Cl(Fe)	2	23.2	–24.6 ± 0.5	2.0 ± 0.2	3.01 ± 0.04	–31.5 ± 1.9
MUV-8-CH_3_(Fe)	2	23.4	–25.2 ± 0.5	2.1 ± 0.2	4.80 ± 0.06	–121 ± 3

a *J* and *g* parameters are extracted by fitting the magnetic data to Lines^21^ and Curély^22^ models for quadratic and hexagonal exchange networks, respectively. The form of the exchange Hamiltonian is .

b **MUV-1(Fe)** data have been taken from ref (13).

Finally, an estimate of the exchange parameters in these 2D antiferromagnetic networks is obtained by fitting the magnetic data to the corresponding theoretical models. To describe the behavior of **MUV-1** derivatives, a model for a quadratic-layer Heisenberg antiferromagnet is used (Lines model;^21^ see SI). This model reproduces in particular the rounded maximum observed in χ in the Mn(II) derivatives (Figure 5b). The exchange parameters for **MUV-8(Fe)** are more difficult to extract since the magnetic network is very complex (see Figure S12). The structure involves two distorted hexagonal lattices, each one formed by two different exchange parameters, *J* and *J*_0_; these two layers are coupled by an additional exchange, *J*_inter_. Since the in-plane distances are in the range 6.0–6.1 Å, while the interplane ones are of 6.2 Å, we could expect the interplanar Fe–Fe exchange interactions to be similar to the in-plane ones. There is no theoretical model available to describe this network. The closest model available involves a single hexagonal lattice of classical spins isotropically coupled (Curély model,^22^ see Section S4). This model could provide a rough estimate of the exchange values in the MUV-8 material. A close fit of the data is obtained using several sets of *J* and *J*_0_ values, indicating a strong correlation between them (see SI). In view of the structure, which shows similar Fe–Fe distances (comprised in between 6.0 and 6.2 Å), we have assumed that both *J* values should be similar and therefore we have set *J* = *J*_0_. Using this model, an antiferromagnetic coupling *J* = *J*_0_ = −25 cm^–1^ is estimated, which is within the range of those obtained for **MUV-1(Fe)** compounds (see Table 1) and may support the above assumption. More precisely, it is slightly stronger (−25 cm^–1^ compared to −23 cm^–1^), which is also in agreement with the higher values observed for *T*_N_ (23 K compared to 20 K). As we can see in this table, the exchange coupling in all the compounds is antiferromagnetic. Interestingly, for a given metallic derivative, the magnetic properties remain unaltered, independent of the type of derivatization on the benzimidazole ligand. Still, these properties change upon changing the metal center (from Fe to Mn and Co for **MUV-1**, for example).

### Magnetic Order by Nanomechanical Resonators

The detection of magnetic order in thin layers of insulating 2D materials is a challenging problem, as it is very difficult to sense such a small amount of material by conventional bulk characterization methods (magnetic or specific heat measurements). In fact, in these 2D materials magnetic ordering has been only detected recently in inorganic magnetic materials by performing Magneto-Optical Kerr Effect measurements at the nanoscale (nanoMOKE),^9^ transport measurements in van der Waals heterostructures,^23−25^ or NV (nitrogen vacancies) magnetometry.^26^ Indirect techniques, such as optical measurement of the second harmonic generation, have also been used to characterize thin layers of inorganic antiferromagnets.^19,27^ Recently, we have demonstrated that the specific heat can be extracted from measuring the temperature dependent nanomechanical resonance frequency of suspended 2D antiferromagnetic membranes.^28^ In particular, it can be shown that the resonance frequency of the fundamental membrane mode, *f*_0_, and the quality factor, *Q*, of a nanomechanical resonator are related to the specific heat, *c*_v_, by the following relations: *c*_v_(*T*) ∝ d(*f*^2^_0_(*T*))/d*T* and *Q*^–1^ ∝ *c*_*v*_(*T*) × *T*. These relations have been used to prove the antiferromagnetic order in the inorganic layers FePS_3_, MnPS_3_, and NiPS_3_.^28^ However, the larger fragility of metal–organic materials to lasers has prevented so far detection of the magnetic order in thin layers of these molecular magnets. In our previous work, we demonstrated that the **MUV-1** family is robust enough to fabricate mechanical resonators and to measure its mechanical properties through laser interferometry.^13^ Taking advantage of this feature—uncommon for a molecular material—we will explore here the possibility to detect magnetic order in these molecular layers using this nanomechanical technique.

**Figure 6:**
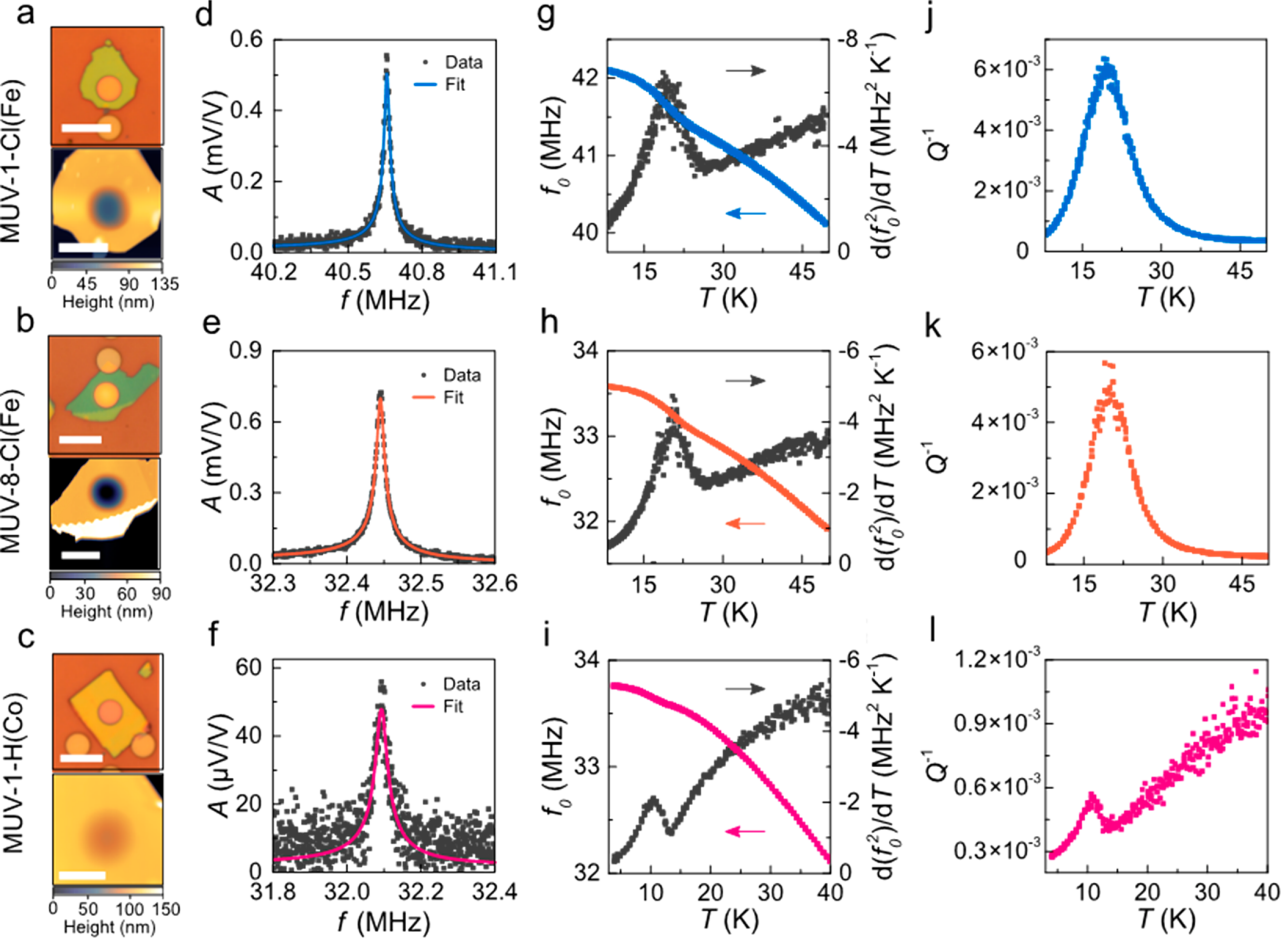
Mechanical resonances of thin MOF membranes. (a–c) **MUV-1-Cl(Fe)**, **MUV-8-Cl(Fe)** and **MUV-1-H(Co)** membranes. (a–c) Top panels: Optical images. Bottom panels: Peak-force atomic force microscopy (AFM) images. (a) Scale bars: Top and bottom panel -10 μm. Membrane thickness *t* = 87.7 ± 0.7 nm and diameter *d* = 5 μm. (b) Scale bars: Top panel - 12 μm. Bottom panel - 6 μm. Membrane thickness *t* = 65.2 ± 1.3 nm and diameter *d* = 6 μm. (c) Scale bars: Top panel - 12 μm. Bottom panel - 6 μm. Membrane thickness *t* = 116.4 ± 1.4 nm and diameter *d* = 6 μm. (d–f) Resonance peaks of the fundamental membrane mode at 40 K. Colored lines - linear harmonic oscillator fit, black dots - measured data. (g–i) Colored dots - resonance frequency *f*_0_ as a function of temperature, black dots - temperature derivative of *f*^2^_0_(*T*) as a function of temperature. (j-l) Mechanical dissipation *Q*^*-1*^ as a function of temperature.

We exfoliate flakes of **MUV-1-Cl(Fe)**, **MUV-8-Cl(Fe)**, and **MUV-1-H(Co)** and transfer these on top of circular cavities (diameter *d* = 5–6 μm) etched in a SiO_2_/Si substrate using deterministic dry viscoelastic stamping to form freestanding nanodrums (see Figure 6a–c).^29^ Due to the large flexibility, low estimated Young’s modulus and, thus, low bending rigidity of these MOF sheets, the suspended flakes behave as membranes even at relatively large thicknesses for a given range of cavity diameters.^13^ The samples are placed in a dry cryostat and cooled down to temperatures of 4 K at a pressure below 10^–6^ mbar. Temperature-dependent mechanical properties of the nanodrums are then investigated using laser interferometry^28^ from 4 to 50 K in the absence of an external magnetic field (see Experimental Section). Figure 6d–f shows resonances of the fundamental membrane mode at 40 K for **MUV-1-Cl(Fe)**, **MUV-8-Cl(Fe)**, and **MUV-1-H(Co)**, respectively (black solid dots).

**Figure 7:**
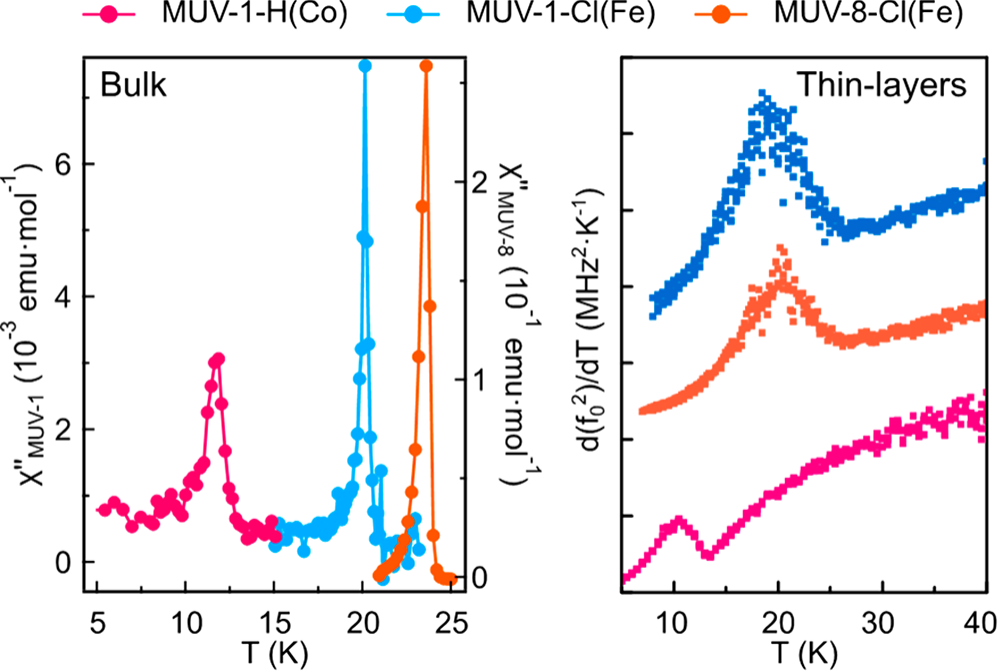
Comparison of the out-of-phase a.c. susceptibility signals for bulk systems (left) and the derivative of *f*_0_^2^(*T*) as a function of temperature for thin layers (right).

We fit the measured resonance peaks to a linear harmonic oscillator model (colored solid lines) and obtain *f*_0_ and *Q*. Substantially large *Q* factors ranging from 1000 to 3500 and high resonance frequencies of 32.1–40.67 MHz at 40 K indicate a high tension in the membranes due to a buildup of the thermal strain. Since the thermal strain in the membranes is related to the thermal expansion coefficient and thus to the specific heat *c*_v_ of the material, we will check if any anomaly is observed in *f*_0_(*T*), related to a phase change.^21^ In Figure 6g–i we plot *f*_0_ (colored filled dots) and the corresponding temperature derivative of *f*^2^_0_ (black filled dots) for all three compounds. The phase transition-related anomaly is well visible as a kink in *f*_0_(*T*) and is even more pronounced in d(*f*^2^_0_ (*T*))/d*T*, which shows a peak at low temperatures that is associated with *T*_N_. As can be seen in Figure 7, the transition temperatures for the thin layers (thickness in the range 65 to 120 nm), extracted from these peaks, are in close agreement with the values determined in Table 1 for the bulk counterparts by a.c. magnetic measurements (**MUV-1-Cl(Fe)**: 19.5 ± 1.0 K, compared to 20.7 K; **MUV-8-Cl(Fe)**: 20.5 ± 1.0 K, compared to 23.2 K; **MUV-1-H(Co)**: 11.0 ± 1.0 K, compared to 12.4 K). The mechanical dissipation *Q*^*—1*^(*T*) also exhibits a local maximum near *T*_N_, as displayed in Figure 6j–l, that can be related to thermoelastic or other more intricate magnetomotive damping mechanism.^28^

## Conclusions

At the intersection between the fields of molecular magnetism and 2D materials, we have exploited the chemical design of layered magnetic coordination polymers based on benzimidazole derivatives with the aim of producing novel 2D molecular-based magnets with novel topologies and tunable magnetic properties. Thus, using a solvent-free synthetic route, we have prepared an isoreticular series of layered materials formed by van der Waals layers with a square 2D magnetic lattice, in which the magnetic properties can be tuned by changing the metallic nodes (from Fe(II) to Co(II) and Mn(II)), while preserving the same crystal structure. In addition, by modifying the organic part (from monosubstituted to disubstituted benzimidazole), it has been possible to change the crystal structure and the magnetic topology from a square lattice to a distorted hexagonal one.

Interestingly, the very weak van der Waals forces between the layers has allowed us to isolate atomically thin layers of micrometer size flakes using a micromechanical exfoliation method. This result can be highly relevant in the field of 2D materials: First, it can offer an alternative way to that provided by solid-state chemistry for the preparation of robust 2D magnets. In fact, the coordination chemistry approach has afforded the isolation of 2D magnetic materials relatively stable in open air, in sharp contrast to the few reported examples of 2D inorganic magnets. Second, the chemical versatility of the method allows novel magnetic topologies to be obtained in two dimensions. The most relevant result in this context has been the isolation of a double layer antiferromagnet exhibiting an unprecedented topology, which is based on the assembly of two hexagonal monolayers through coordination bonds. Third, the employed solvent-free methodology is compatible with chemical vapor deposition techniques, thus allowing the future scalability of these 2D magnetic MOFs.

Nanomechanical resonators made from the MOFs have been used to detect the magnetic order in thin layers of these 2D molecular-based antiferromagnets. Due to the strong coupling between mechanical motion and magnetic order, this technique has provided additional confirmation of the critical temperatures in these thin layer systems. The use of this nanomechanical approach for detecting magnetic order in molecular materials is believed to be limited by their inherent instability to the exposure of the laser beam, which can damage the suspended membranes. Still, the layered MOFs studied have shown to be stable enough to be measured using this technique. In the present case, this has allowed the study of the magnetic order in membranes with layer thickness down to 65 nm, although this thickness is still far from the 2D limit (1–2 nm). More robust and thinner membranes will be prepared in the future taking advantage of the possibility of fabricating van der Waals heterostructures using a deterministic method.^30^ For example, by combining ultrathin layers of these MOFs with atomically thin layers of an inorganic material (h-BN layer, for instance), we expect to overcome this limitation. This will open interesting possibilities in 2D physics. Also, the tuning of *T*_N_ with the dimensionality of the system (number of magnetic layers), or with the strain of the membrane induced by an external stimulus (like an electrostatic gate voltage),^31^ could in future be realized.

## Experimental Section

All reagents were commercially available and were used without further purification.

### Synthesis of MUV-1-X(M^II^)

A metallic source (0.16 mmol) and benzimidazole derivate (0.34 mmol) were combined and sealed under vacuum in a layering tube (4 mm diameter). The mixture was heated at 150 °C for 4 days to obtain crystals suitable for X-ray single-crystal diffraction. The product was allowed to cool to room temperature, and the layering tube was then opened. The unreacted precursors were extracted with acetonitrile and benzene, and the main compound was isolated as crystals (yield 60%). Phase purity was established by X-ray powder diffraction.

### Synthesis of MUV-8-X(Fe)

Ferrocene (30 mg, 0.16 mmol) and 5,6-dichlorobenzimidazole (64 mg, 0.34 mmol) or 5,6-dimethylbenzimidazole (50 mg, 0.34 mmol) were combined and sealed under vacuum in a layering tube (4 mm diameter). The mixture was heated at 250 °C for 3 days to obtain colorless crystals suitable for X-ray single-crystal diffraction. The product was allowed to cool to room temperature, and the layering tube was then opened. The unreacted precursors were extracted with acetonitrile and benzene, and the main compound was isolated as colorless crystals (yield 60%). Phase purity was established by X-ray powder diffraction.

### Single Crystal X-ray Diffraction

X-ray data for compounds **MUV-1-H(Co)** and **MUV-8-Cl(Fe)** were collected at a temperature of 120 K using a Mo-kα radiation on a Rigaku Supernova diffractometer equipped with an Oxford Cryosystems nitrogen flow gas system. X-ray data for compound **MUV-1-Cl(Mn)** were collected at a temperature of 100 K using a Cu-kα radiation on a Rigaku FR-X diffractometer equipped with an Oxford Cryosystems nitrogen flow gas system. Details on the crystal structure determination and refinements can be found in Section S2 of the Supporting Information.

### Magnetic Properties

Variable-temperature (2–300 K) direct current (d.c.) magnetic susceptibility measurements were carried out in an applied field of 1.0 kOe, and variable field magnetization measurements up to ±5 T, at 2.0 K. The susceptibility data were corrected from the diamagnetic contributions as deduced by using Pascal’s constant tables. Variable-temperature (16–23 K) alternating current (ac) magnetic susceptibility measurements in a ±4.0 G oscillating field at frequencies in the range 1–997 Hz were carried out in a zero d.c. field. All the measurements were performed with a SQUID magnetometer (Quantum Design MPMS-XL-5 and MPMS-XL-7).

### Laser Interferometry

The motion of the nanodrums was measured using a laser interferometry setup, similar to the one reported in ref (28). The sample is mounted on an xyz piezomotive nanopositioning stage inside the chamber of a Montana Cryostation s50 dry optical cryostat. A blue diode laser (λ = 405 nm), which is power-modulated by a Vector Network Analyzer (VNA), is focused at the center of the membrane and used to optothermally excite the membrane into motion at a given frequency. A red He–Ne laser (λ = 632 nm) is used to read out the vibrations of the nanodrum, which are analyzed using a homodyne detection scheme and processed by the VNA. The laser spot diameter is in the order of 1 μm. It is checked that the resonance frequency changes due to laser heating are insignificant for all membranes.

### Peak-Force AFM

All peak-force AFM data are acquired at a constant 50 nN of applied peak force using a cantilever with a spring constant *k* = 22.8 N m^—1^ on Bruker Dimension FastScan AFM. The thickness of the sample is estimated taking the average of 3 to 5 profile scans.

## Supporting Information Available

Additional experimental details, materials, and methods (PDF)

## Accession Codes

CCDC 2068293–2068295 contain the supplementary crystallographic data for this paper. These data can be obtained free of charge via www.ccdc.cam.ac.uk/data_request/cif, or by emailing data_request@ccdc.cam.ac.uk, or by contacting The Cambridge Crystallographic Data Centre, 12 Union Road, Cambridge CB2 1EZ, UK; fax: +44 1223 336033.
