# Supplementary material for: Chemical Design and Magnetic Ordering in Thin Layers of 2D Metal–Organic Frameworks (MOFs)
Source: J Am Chem Soc. 2021 Nov 1;143(44):18502–10. doi: 10.1021/jacs.1c07802 (PMC8587609; doi:10.1021/jacs.1c07802)
Supplement: Supplementary file 1 — ja1c07802_si_001.pdf [file ja1c07802_si_001.pdf]

Chemical design and magnetic ordering in thin layers of 2D MOFs

Javier López-Cabrelles, [$1] Samuel Mañas-Valero,[$1] Iñigo J. Vitórica-Yrezábal,[2] Makars Šiškins,[3] Martin Lee,[3] Peter G. Steeneken,[3,4] Herre S. J. van der Zant,[3] Guillermo Mínguez Espallargas*[1] and Eugenio Coronado*[1]

[1]Instituto de Ciencia Molecular (ICMol), Universidad de Valencia, c/Catedrático José Beltrán, 2, 46980 Paterna, Spain.

[2]School of Chemistry, University of Manchester, Manchester, UK.

[3]Kavli Institute of Nanoscience, Delft University of Technology, Lorentzweg 1, 2628 CJ, Delft, The Netherlands.

[4]Department of Precision and Microsystems Engineering, Delft University of Technology, Mekelweg 2, 2628 CD, Delft, The Netherlands.

[$]Equally contributed

Contents

S1. Materials and synthesis 2

Synthesis of MUV-1-Cl(Co) [$Co(bim)_2$] 2

Synthesis of MUV-1-Cl(Co) [$Co(bimCl)_2$] 2

Synthesis of MUV-1-Cl(Mn) [$Mn(bim)_2$] 2

Synthesis of MUV-1-Cl(Mn) [$Mn(bimCl)_2$] 3

Synthesis of MUV-1-H(Zn) [$Zn(bim)_2$] 3

Synthesis of MUV-1-Cl(Zn) [$Zn(bimCl)_2$] 4

Synthesis of MUV-8-Cl(Fe) [$Fe(bimCl_2)_2$] 4

Synthesis of MUV-8-CH_3(Fe) [$Fe(bim(CH_3)_2)_2$] 4

S2. Structure of MUV-1-X(M(II)) and MUV-8-X(Fe) family 5

S2.1 Data Collection 5

S2.2 Crystal structure determinations and refinements 5

S3. Characterization of MUV-1-X(M(II) = Co, Mn, Zn) family and MUV-8-X(Fe) 9

S3.1 X-Ray powder diffraction 9

S3.2 Thermal stability 11

S3.3 Scanning electronic microscope images 13

S4. Magnetic properties 17

S4.1 SQUID measurements and magnetic models 17

S4.2 Electron Paramagnetic Resonance measurements 34

S5. Two-dimensional approach and characterization 36

S5.1 Atomic Force Microscopy of the few layers of MUV-1-Cl(Co), MUV-1-H(Co), MUV-8-Cl(Fe) and MUV-8-CH_3(Fe) 36

S5.2. Raman spectroscopy in bulk and two-dimensional layers of MUV-1-X(Co) and MUV-8-X(Fe). 40

S6. Bibliography 45

S1. Materials and synthesis

Synthesis. The compounds were prepared adapting a previously described method for the preparation of iron imidazolates. All reagents and solvents were commercially available and used without further purification.

Synthesis of MUV-1-Cl(Co) [Co(bim)$_2$]

Cobaltocene (30 mg, 0.16 mmol) and benzimidazole (40 mg, 0.34 mmol) were combined and sealed under vacuum in a layering tube (4 mm diameter). The mixture was heated at 150 °C for 4 days to obtain purple crystals suitable for X-ray single-crystal diffraction. The product was allowed to cool to room temperature, and the layering tube was then opened. The unreacted precursors were extracted with acetonitrile and benzene, and the main compound was isolated as purple crystals. Phase purity was established by X-ray powder diffraction.

Synthesis of MUV-1-Cl(Co) [Co(bimCl)$_2$]

Cobaltocene (30 mg, 0.16 mmol) and 5-chlorobenzimidazole (49 mg, 0.34 mmol) were combined and sealed under vacuum in a layering tube (4 mm diameter). The mixture was heated at 150 °C for 4 days to obtain purple crystals suitable for X-ray single-crystal diffraction. The product was allowed to cool to room temperature, and the layering tube was then opened. The unreacted precursors were extracted with acetonitrile and benzene, and the main compound was isolated as purple crystals. Phase purity was established by X-ray powder diffraction.

Synthesis of MUV-1-Cl(Mn) [Mn(bim)$_2$]

Bis(tetramethylcyclopentadienyl)manganese(II) (47.5 mg, 0.16 mmol) and benzimidazole (40 mg, 0.34 mmol) were combined and sealed under vacuum in a layering tube (4 mm diameter). The mixture

was heated at 250 °C for 3 days to obtain colourless crystals suitable for X-ray single-crystal diffraction. The product was cooled down naturally to room temperature, and the layering tube was then opened. The unreacted precursors were extracted with acetonitrile and benzene, and the main compound was isolated as colourless crystals. Phase purity was established by X-ray powder diffraction.

Synthesis of MUV-1-Cl(Mn) [Mn(bimCl)$_2$]

Bis(tetramethylcyclopentadienyl)manganese(II) (47.5 mg, 0.16 mmol) and 5-chlorobenzimidazole (49 mg, 0.34 mmol) were combined and sealed under vacuum in a layering tube (4 mm diameter). The mixture was heated at 250 °C for 3 days to obtain colourless crystals suitable for X-ray single-crystal diffraction. The product was allowed to cool to room temperature, and the layering tube was then opened. The unreacted precursors were extracted with acetonitrile and benzene, and the main compound was isolated as colourless crystals. Phase purity was established by X-ray powder diffraction.

Synthesis of MUV-1-H(Zn) [Zn(bim)$_2$]

Bis(2,2,6,6-tetramethyl-3,5-heptanedionato) zinc(II) (69.1 mg, 0.16 mmol) and benzimidazole (40 mg, 0.34 mmol) were combined and sealed under vacuum in a layering tube (4 mm diameter). The mixture was heated at 180 °C for 4 days to obtain colourless crystals suitable for X-ray single-crystal diffraction. The product was allowed to cool to room temperature, and the layering tube was then opened. The unreacted precursors were extracted with acetonitrile and benzene, and the main compound was isolated as colourless crystals. Phase purity was established by X-ray powder diffraction.

Synthesis of MUV-1-Cl(Zn) [Zn(bimCl)$_2$]

Bis(2,2,6,6-tetramethyl-3,5-heptanedionato) zinc(II) (69.1 mg, 0.16 mmol) and 5-chlorobenzimidazole (49 mg, 0.34 mmol) were combined and sealed under vacuum in a layering tube (4 mm diameter). The mixture was heated at 180 °C for 4 days to obtain colourless crystals suitable for X-ray single-crystal diffraction. The product was allowed to cool to room temperature, and the layering tube was then opened. The unreacted precursors were extracted with acetonitrile and benzene, and the main compound was isolated as colourless crystals. Phase purity was established by X-ray powder diffraction.

Synthesis of MUV-8-Cl(Fe) [Fe(bimCl$_2$)$_2$]

Ferrocene (30 mg, 0.16 mmol) and 5,6-dichlorobenzimidazole (64 mg, 0.34 mmol) were combined and sealed under vacuum in a layering tube (4 mm diameter). The mixture was heated at 250 °C for 3 days to obtain colourless crystals suitable for X-ray single-crystal diffraction. The product was allowed to cool to room temperature, and the layering tube was then opened. The unreacted precursors were extracted with acetonitrile and benzene, and the main compound was isolated as colourless crystals. Phase purity was established by X-ray powder diffraction.

Synthesis of MUV-8-CH$_3$(Fe) [Fe(bim(CH$_3$)$_2$)$_2$]

Ferrocene (30 mg, 0.16 mmol) and 5,6-dimethylbenzimidazole (50 mg, 0.34 mmol) were combined and sealed under vacuum in a layering tube (4 mm diameter). The mixture was heated at 250 °C for 3 days to obtain colourless crystals suitable for X-ray single-crystal diffraction. The product was allowed to cool to room temperature, and the layering tube was then opened. The unreacted precursors were extracted with acetonitrile and benzene, and the main compound was isolated as colourless crystals (yield 60 %). Phase purity was established by X-ray powder diffraction.

S2. Structure of MUV-1-X(M(II)) and MUV-8-X(Fe) family

S2.1 Data Collection

X-Ray data for compound **MUV-1-H(Co) and MUV-8-Cl(Fe)** were collected at a temperature of 120 K using a Mo-kα radiation on a Rigaku Supernova diffractometer equipped an Oxford Cryosystems nitrogen flow gas system. X-Ray data for compound **MUV-1-Cl(Mn)** were collected at a temperature of 100 K using a Cu-kα radiation on a Rigaku FR-X diffractometer equipped an Oxford Cryosystems nitrogen flow gas system. Data were measured using CrisAlisPro suite of programs.

S2.2 Crystal structure determinations and refinements

X-Ray data were processed and reduced using CrysAlisPro suite of programmes. Absorption correction was performed using empirical methods (SCALE3 ABSPACK) based upon symmetry-equivalent reflections combined with measurements at different azimuthal angles. The crystal structure was solved and refined against all F^2 values using the SHELXL and Olex 2 suite of programmes.[1,2] All atoms were refined anisotropically. Hydrogen atoms were placed in the calculated positions. Pyridine moieties in crystal **MUV-1-H(Co)** were disordered and modelled over two positions. Ferrocene molecules were disordered and modelled over two positions in **MUV-8-Cl(Fe)**. The atomic displacement parameters were also restrained using SHELXs SIMU and RIGU commands.

Crystals of **MUV-1-H(Co)** and **MUV-1-Cl(Mn)** were found to be modulated commensurate with a q vector (½, ½, 0). Despite that the coordination polymers are intrinsically chiral; the centrosymmetric space group C2/c was found as result of the racemic distribution of the disordered layers.

CCDC 2068293-2068295 contains the supplementary crystallographic data for this paper. These data can be obtained free of charge *via* www.ccdc.cam.ac.uk/conts/retrieving.html (or from the Cambridge Crystallographic Data Centre, 12 Union Road, Cambridge CB21EZ, UK; fax: (+44)1223-336-033; or deposit@ccdc.cam.ac.uk).

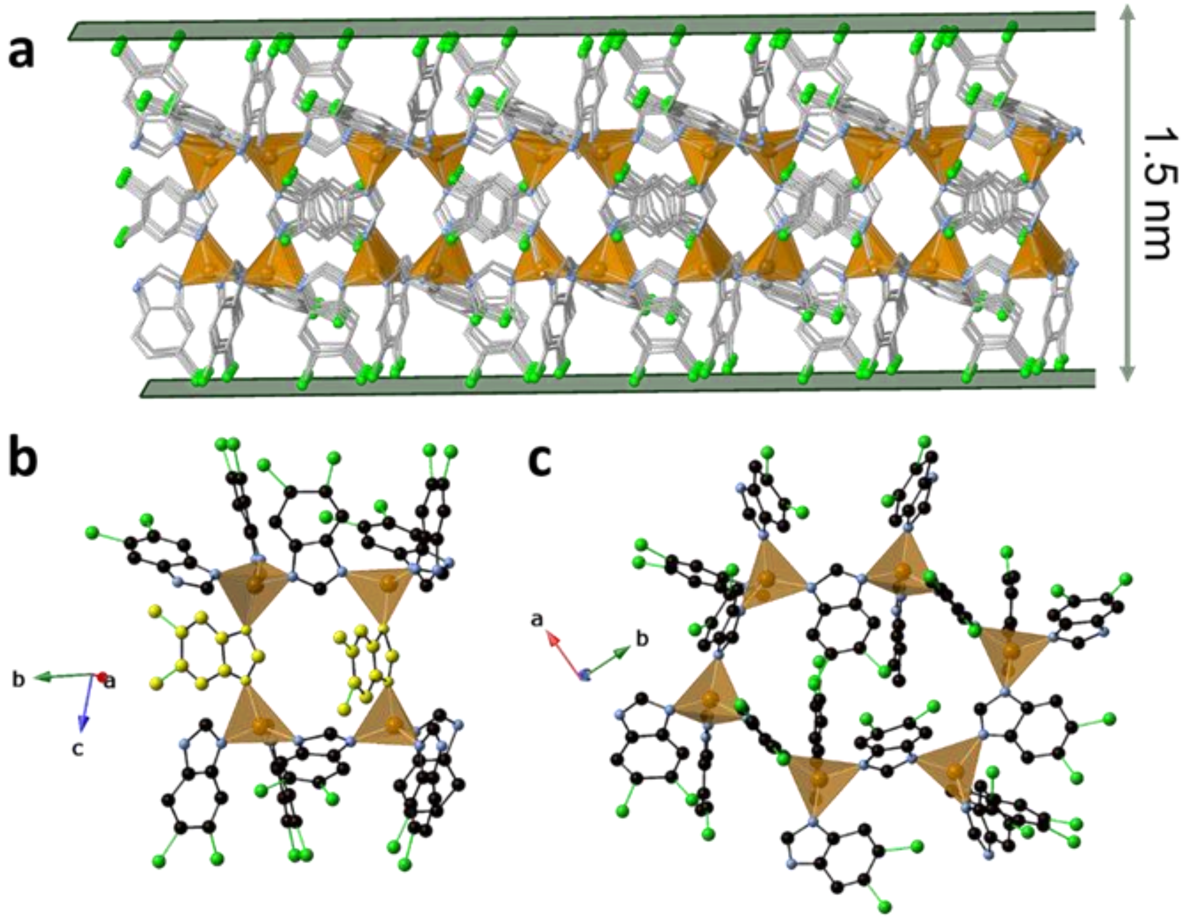

Figure S1. a) Crystal structure of the **MUV-8-Cl(Fe)**, composed by Fe(II) centers in a tetrahedral environment, forming extended layers in the *ab* plane. b) The connectivity in one sheet, three ligands connecting in *ab* plane, and the fourth ligand (in yellow) connecting the two layers of iron centers (the Fe bilayer). c) The distorted hexagonal lattice in the perpendicular *ab* plane view.

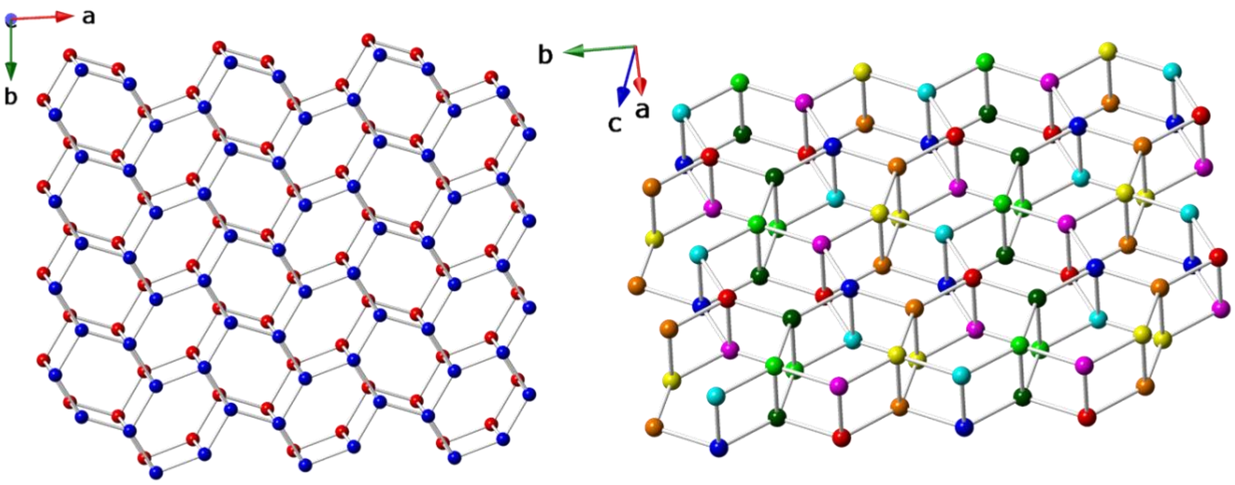

Figure S2. Crystal structure of **MUV-8-Cl(Fe).** Bilayered structure for a monolayer, similar to AB stacking. Only iron centres are represented (C, N, H, and Cl atoms are omitted) (left). Seven different iron centres in a monolayer. Different colours have been used to differentiate one from the other. Only iron centres are represented (C, N, H, and Cl atoms are omitted) (right).

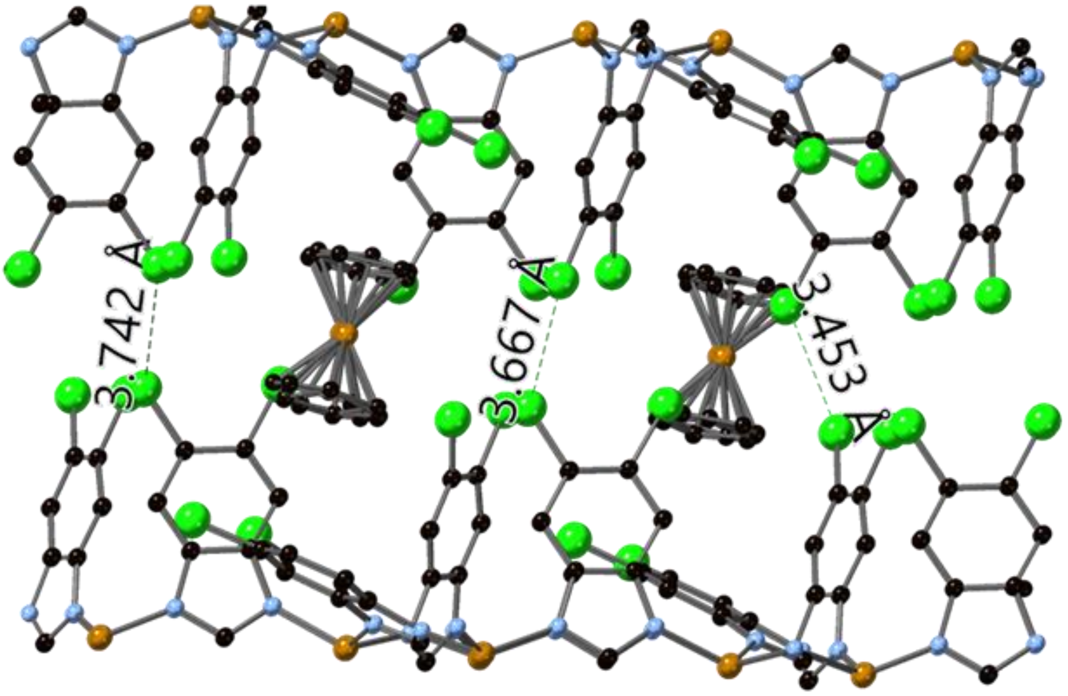

Figure S3. Crystal structure of **MUV-8-Cl(Fe)**. with the location of ferrocene molecules in the interlayer space.

Table S1. Crystallographic information for compounds **MUV-1-X** and **MUV-8-Cl(Fe).**

Identification code	**MUV-8-Cl(Fe)**	**MUV-1-H(Co)**	**MUV-1-Cl(Mn)**
Empirical formula	$C_{66}H_{34}Cl_{16}Fe_5N_{16}$	$C_{14}H_{10}CoN_4$	$C_{14}H_8Cl_2MnN_4$
Formula weight	1897.54	293.19	358.08
Temperature/K	120.00(10)	119.8(4)	99.8(8)
Crystal system	triclinic	monoclinic	monoclinic
Space group	P-1	C2/c	C2/c
a/Å	10.4429(6)	8.0785(11)	8.3680(2)
b/Å	17.5917(9)	8.0783(12)	8.3838(2)
c/Å	21.340(2)	19.258(3)	20.9429(6)
α/°	100.270(7)	90	90
β/°	101.992(7)	96.159(13)	95.897(2)
γ/°	90.045(4)	90	90
Volume/Å^3	3770.4(5)	1249.5(3)	1461.49(6)
Z	2	4	4
ρ_{calc}g/cm^3	1.671	1.559	1.627
μ/mm^{-1}	1.559	1.362	10.684
F(000)	1888.0	596.0	716.0
Crystal size/mm^3	0.1 × 0.06 × 0.05	0.1 × 0.1 × 0.1	0.03 × 0.02 × 0.02
Radiation	Mo Kα (λ = 0.71073)	Mo Kα (λ = 0.71073)	Cu Kα (λ = 1.54184)
2Θ range for data collection/°	3.344 to 50.7	7.154 to 50.038	8.49 to 151.912
Index ranges	$-12 \leq h \leq 12, -21 \leq k \leq 21, -25 \leq l \leq 25$	$-9 \leq h \leq 9, -8 \leq k \leq 9, -3 \leq l \leq 22$	$-10 \leq h \leq 10, -10 \leq k \leq 10, -25 \leq l \leq 23$
Reflections collected	13813	1108	8674
Independent reflections	13813 [R_{int} = 0.1984, R_{sigma} = 0.2133]	1108 [R_{int} = 0.101, R_{sigma} = 0.0766]	1525 [R_{int} = 0.0450, R_{sigma} = 0.0334]
Data/restraints/parameters	13813/1393/1029	1108/222/152	1525/0/168
Goodness-of-fit on F^2	1.041	1.263	1.036
Final R indexes [I>=2σ (I)]	R_1 = 0.0967, wR_2 = 0.2102	R_1 = 0.1193, wR_2 = 0.2820	R_1 = 0.0400, wR_2 = 0.1008
Final R indexes [all data]	R_1 = 0.1991, wR_2 = 0.2647	R_1 = 0.1262, wR_2 = 0.2861	R_1 = 0.0413, wR_2 = 0.1025
Largest diff. peak/hole / e Å^{-3}	1.70/-1.14	1.55/-3.29	0.35/-0.48

[a] $R1(F) = \Sigma(|F_o| - |F_c|)/\Sigma|F_o|$; [b] $wR^2(F^2) = [\Sigma w(F_o^2 - F_c^2)^2/\Sigma wF_o^4]^{½}$; [c] $S(F^2) = [\Sigma w(F_o^2 - F_c^2)^2/(n + r - p)]^{½}$

S3. Characterization of MUV-1-X(M(II) = Co, Mn, Zn) family and MUV-8-X(Fe)

Polycrystalline samples of **MUV-1-H(Co), MUV-1-Cl(Co), MUV-1-H(Mn), MUV-1-Cl(Mn), MUV-8-Cl(Fe)**, **MUV-8-CH$_3$(Fe)**were lightly ground in an agate mortar and pestle and used to fill a 0.5 mm borosilicate capillariy that was mounted and aligned on an Empyrean PANalytical powder diffractometer, using Cu Kα radiation (λ = 1.54056 Å). Two repeated measurements were collected at room temperature (2θ = 5−40 °) and merged in a single diffractogram. Thermogravimetric analysis of **MUV-1-H(Co)** and **MUV-1-Cl(Co)** were carried out with a Mettler Toledo TGA/SDTA851e/SF/1100 apparatus in the 25–600 °C temperature range under a 5°C·min^{-1} scan rate and an air flow of 30 mL·min^{-1}. Scanning Electronic Micrographs and atomic composition of bulk samples was estimated by electron probe microanalysis (EPMA) performed in a Philips SEM XL30 equipped with an EDAX microprobe and images were recorded in a Hitachi S-4800. Raman spectra were acquired with a micro-Raman (model XploRA ONE from Horiba, Kyoto, Japan) with a grating of 2400 gr/mm, slit of 50 µm, and hole of 500 µm. The employed wavelengths were 532 nm, 638 nm, and 785 nm. The power density of the laser used for spectra measured at 532 nm was 5.25 mW/µm^2 (bulk crystals) and 170 µW/µm^2 (thin-layers), for spectra measured at 638 nm it was 7.58 mW/µm^2 (bulk crystals), and for those spectra measured at 785 nm it was 7.2 mW/µm^2 (bulk crystals).

S3.1 X-Ray powder diffraction

In Figure S4-S5 the experimental X-ray powder diffraction patterns (in color) are summarized and compared with the theoretical diffractogram. It is worth to mention the high correlation between the experimental and the theoretical diffractograms.

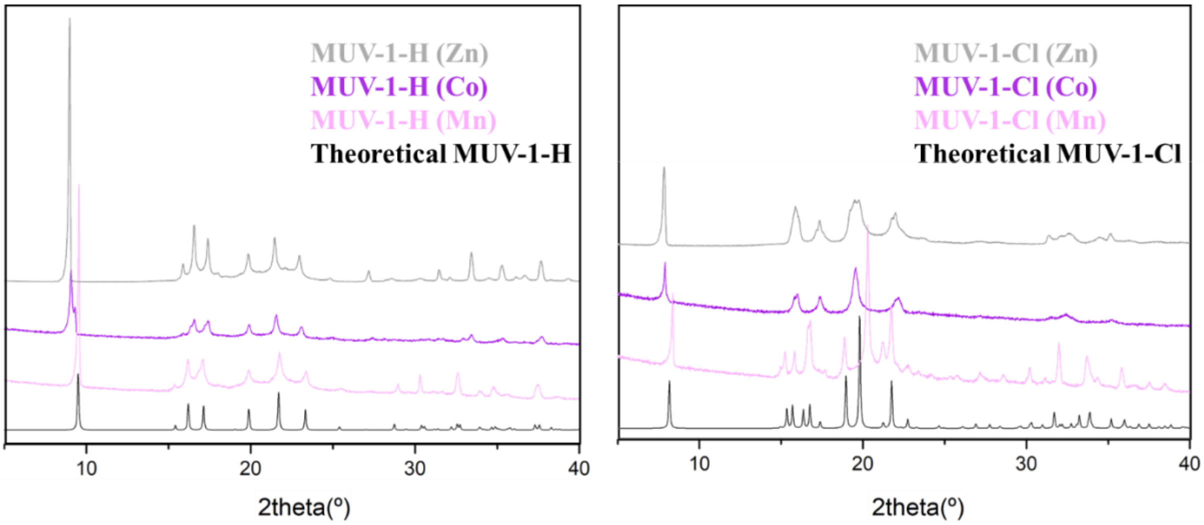

Figure S4. X-ray powder patterns of **MUV-1-H(M(II))** and **MUV-1-Cl(M(II).** The experimental patterns are shown in pink (Mn(II)), purple (Co(II)) and grey (Zn(II)) for the respective compounds, and the calculated pattern from single crystal data are shown in black for all compounds.

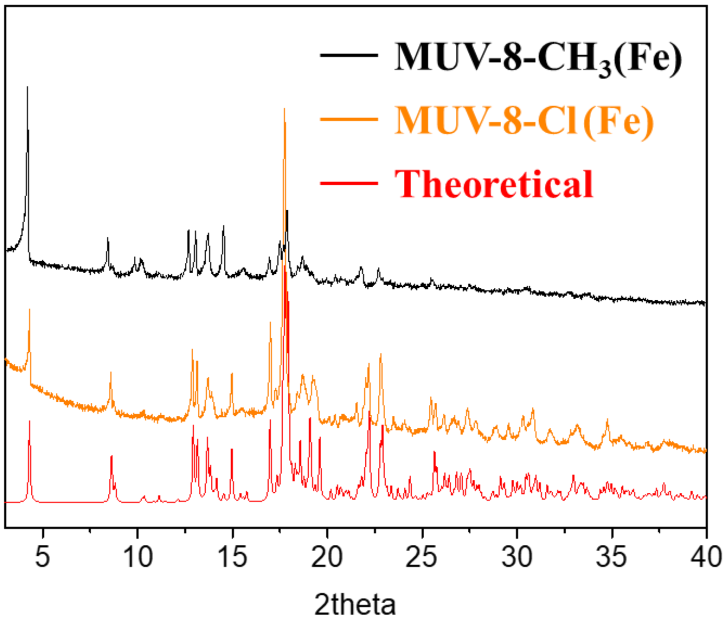

Figure S5. X-ray powder patterns of **MUV-8-Cl(Fe)** and **MUV-8-CH_3(Fe)** .The experimental patterns are shown in orange and black for the respective compounds, and the calculated pattern from single crystal data are shown in red for all compounds.

S3.2 Thermal stability

The thermal stability of all the compounds was determined by thermal gravimetric analysis at a heating rate of 20 ºC min^{-1}.

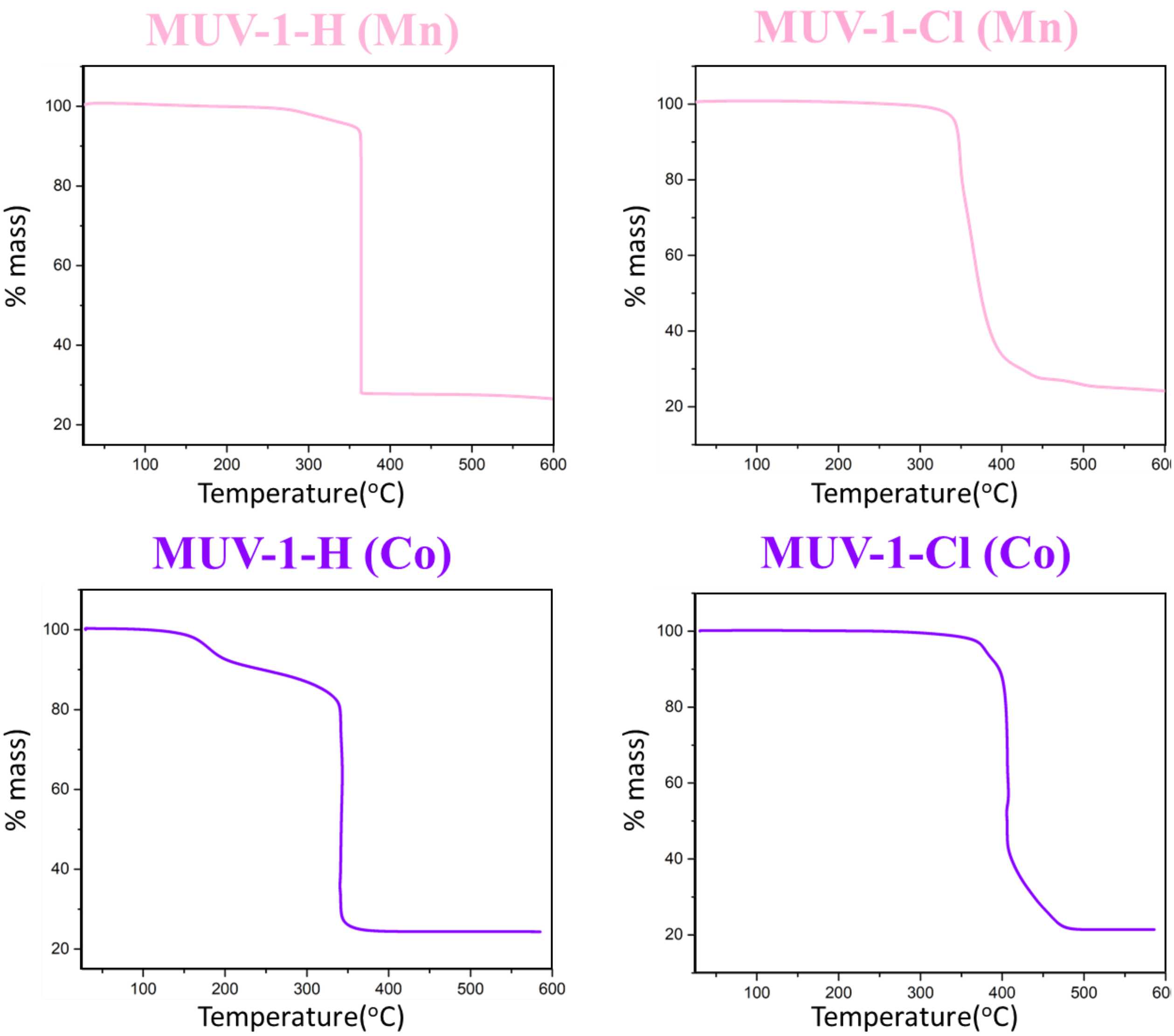

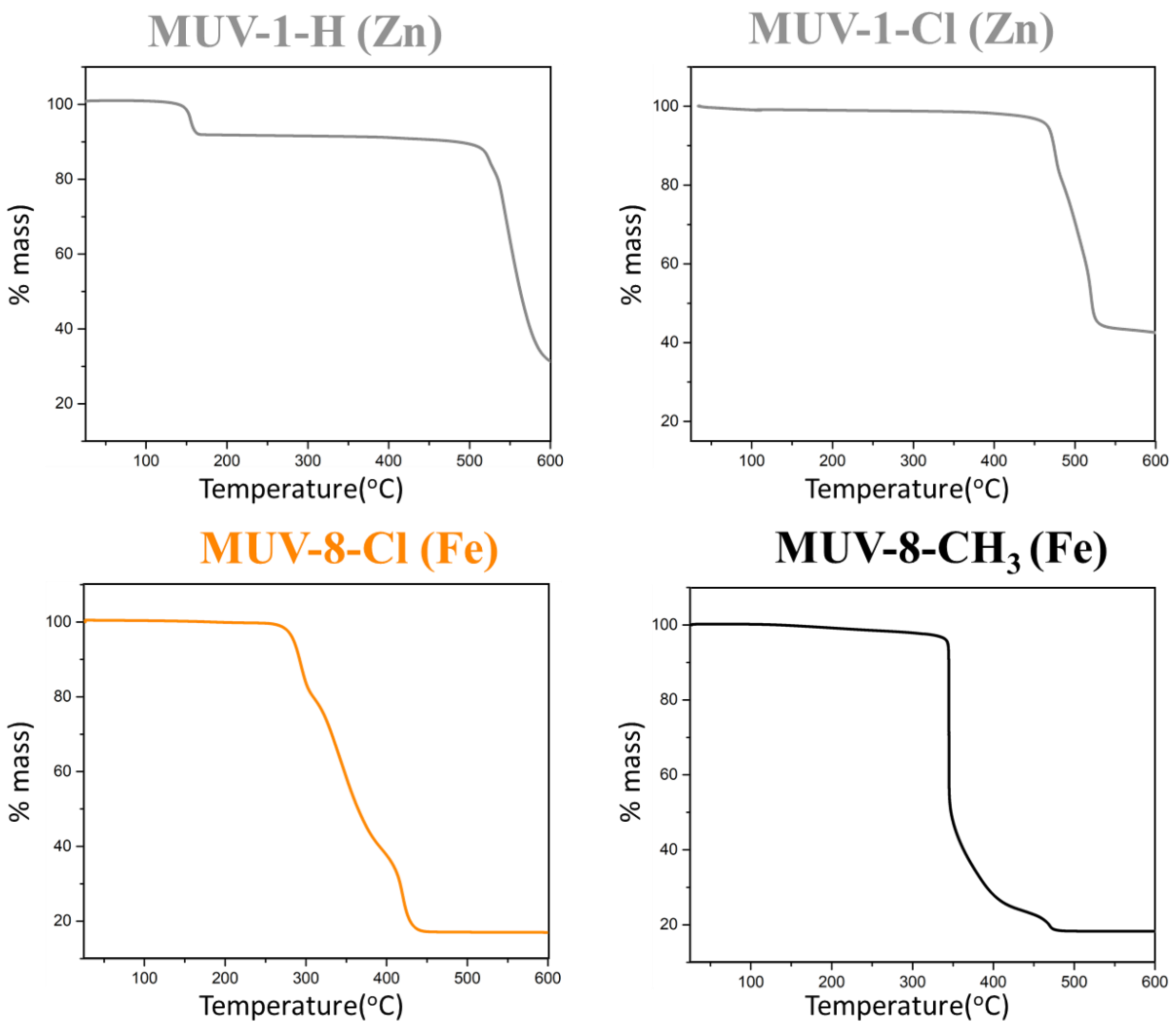

Figure S6. Thermal gravimetric analysis (TGA) of bulk crystals of **MUV-1-H(Co), MUV-1-Cl(Co)**, **MUV-1-H(Mn), MUV-1-Cl(Mn), MUV-1-H(Zn), MUV-1-Cl(Zn), MUV-8-Cl(Fe)** and **MUV-8-CH3(Fe)** at a heating rate of 20 °C min^{-1}.

S3.3 Scanning electronic microscope images

The layered structure of **MUV-1-Cl(Co), MUV-1-H(Co), MUV-1-Cl(Mn) and MUV-1-H(Mn),** was clearly identified by SEM images. As it can be seen in Figure S7-S10, well-defined rectangular edges are the main characteristic of these compounds.

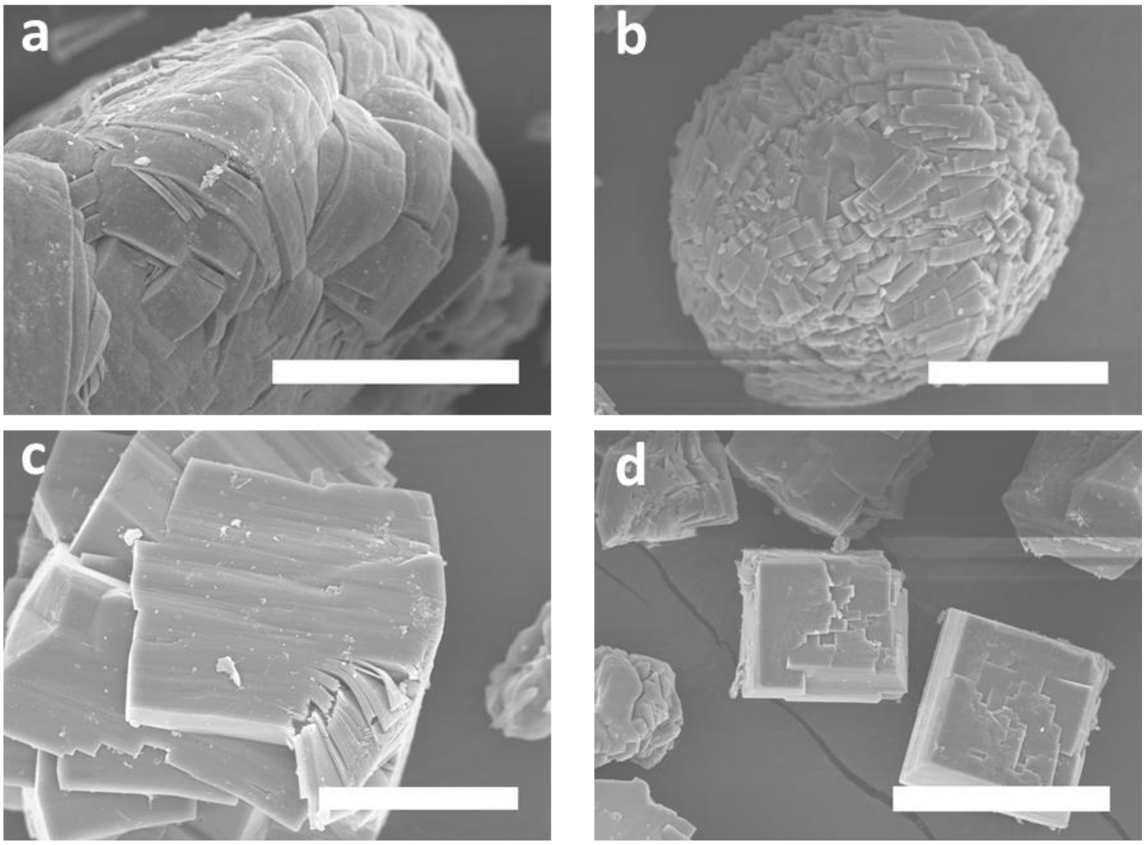

Figure S7. Scanning electron micrograph of bulk-type **MUV-1-Cl(Co)** (a,b) and **MUV-1-H(Co)** (c,d). Scale bar is a) 50 µm b) 20 µm c) 20 µm d) 40 µm.

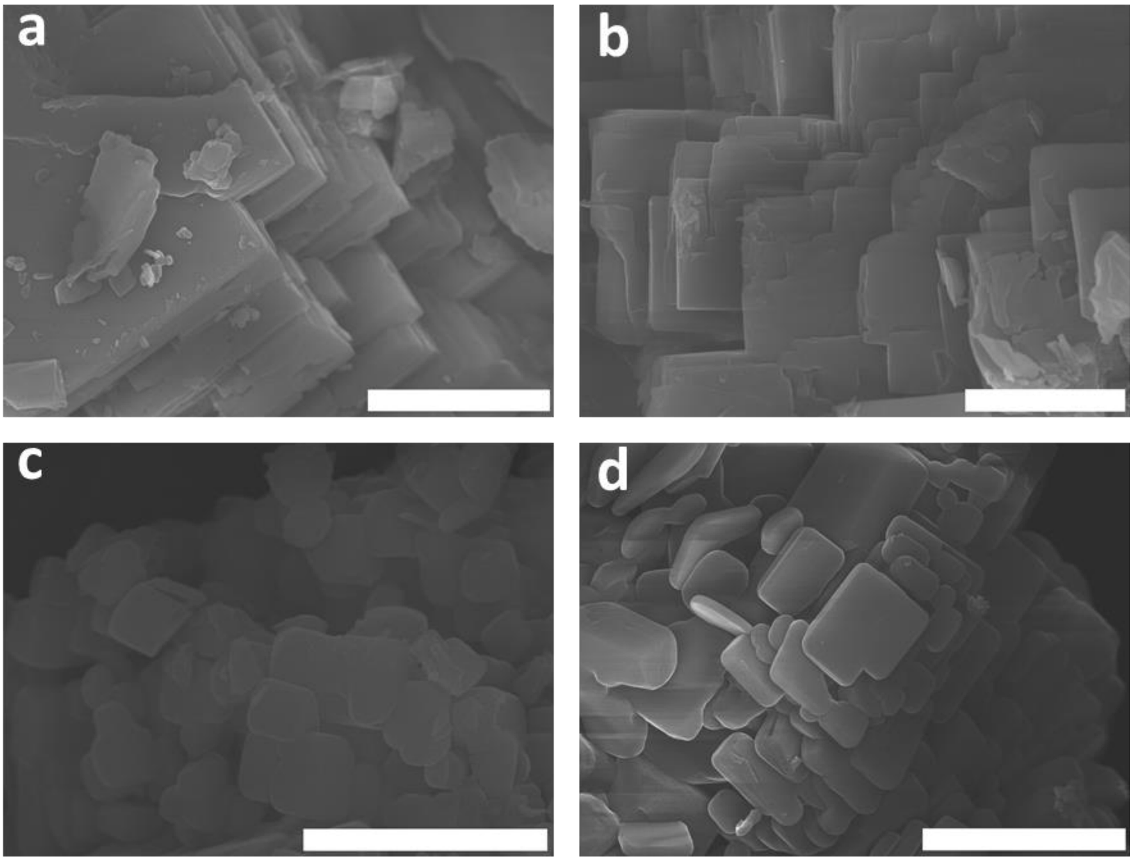

Figure S8. Scanning electron micrograph of bulk-type **MUV-1-Cl(Mn)** (a,b) and **MUV-1-H(Mn)** (c,d)**.** Scale bar is a-b) 10 µm c-d) 5 µm.

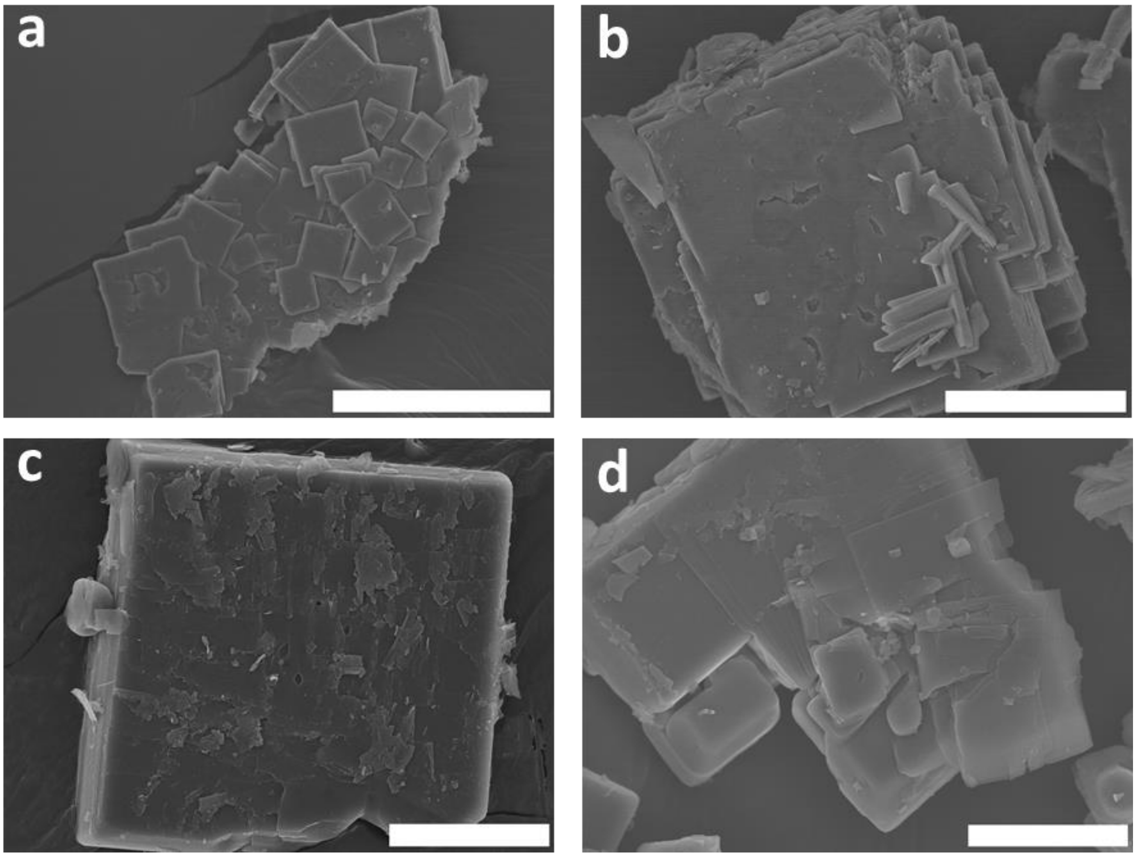

Figure S9. Scanning electron micrograph of bulk-type **MUV-1-Cl(Zn)** (a,b) and **MUV-1-H(Zn)** (c,d)**.** Scale bar is a) 40 µm b) 20 µm c-d) 10 µm.

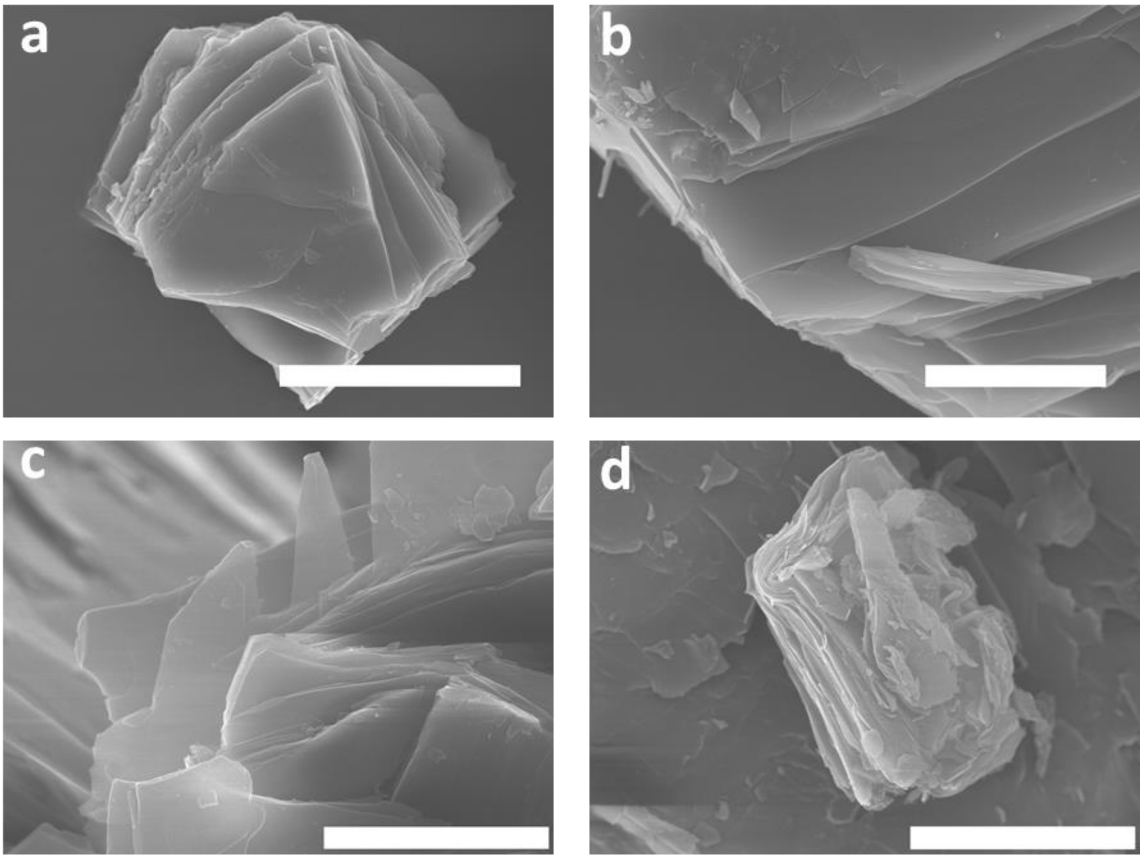

Figure S10. Scanning electron micrograph of bulk-type **MUV-8-Cl(Fe)** (a,b) and **MUV-8-CH_3(Fe)** (c,d). Scale bar is a) 30 µm b-d) 10.

S4. Magnetic properties

S4.1 SQUID measurements and magnetic models

Magnetic measurements are performed with a SQUID magnetometer (Quantum Design MPMS-XL-5). Direct current (dc) magnetic susceptibility is measured with an applied field of 1000 Oe whereas alternating current (ac) measurements are performed at different frequencies in a zero dc field and an oscillating 4 G field. Electron Paramagnetic Resonance (EPR) spectroscopy measurements were recorded with a Bruker ELEXYS E580 spectrometer operating in the Q band (34 GHz).

The magnetic measurements for all the different compounds are shown in Figure S13-S14 (magnetic susceptibility, χ, versus temperature), Figure S16 (χT vs. T), Figure S.17 (χ^{-1} vs. T), Figure S18 (magnetization vs. external field at 2 K) and Figure S19-S21 (a.c. χ vs. T).

The temperature dependence of the dc susceptibility increases while cooling down until a broad maximum is reached. Below this temperature, there is a sharp increase of χ –attributed to spin-canting and that it is accompanied by a sharp peak in the ac measurements– for all the compounds except for the manganese systems followed by a decrease of the susceptibility due to the existent antiferromagnetic interactions. In some compounds, it is observed an enhancement of the susceptibility at the lowest temperatures due to the presence of some paramagnetic impurities. The Néel temperature is assigned as the point where the out-of-phase component of the a.c. susceptibility differs from 0 in the case of the systems with spin-canting and following the Fisher criteria (maximum in $\frac{\partial(\chi T)}{\partial T}$) in the case where the canting is absent (**MUV-1(Mn)**).[3,4]

In the case of the **MUV-1** family, the magnetic coupling constant, J, is determined by fitting the susceptibility versus temperature curves with the Lines expression[16] for a quadratic-layer antiferromagnet based on the following Hamiltonian: $\mathrm{H} = -\sum_{i,j} J S_i \cdot S_j$, where $J < 0$ indicates antiferromagnetic correlations.

$$\chi = -\frac{Ng^2\mu_B^2}{J}\left(3\frac{k_BT}{JS(S+1)} + \sum_{n=1}^{6}\frac{C_n}{\left(\frac{k_BT}{JS(S+1)}\right)^{n-1}}\right)^{-1}$$

where J is the exchange constant, k_B is the Boltzmann constant, S is the spin, N is the Avogadro´s number, g is the Landé factor, μ_B is the Bohr magneton and C_n are coefficients of the Lines expansion (see ref. [16]).

Regarding the **MUV-8-X(Fe)** compounds, the magnetic properties are more challenging to model due to their complex topology. As an initial ansatz, we approximate it to a 2D Heisenberg honeycomb lattice with antiferromagnetic correlations between classical spins, as developed by Curély *et al.*[17] This model adapts the two-dimensional Heisenberg classical square lattice case to the hexagonal one by considering two different exchange constants, J and J_0. The former is related to vertical couplings while the latter takes into account the horizontal ones,[17] as sketched in Figure S11. In the limiting cases, the model yields to the formation of chains ($J_0 = 0$) or dimers ($J = 0$). The model starts with a square lattice with four different exchange constants between first neighbor atoms (left panel in Figure S11). By setting one of them to zero and equaling the perpendicular ones ($J'_0 = 0$ and $J = J_1$ in the middle panel of Figure S11), a hexagonal arrangement is obtained, which is more evident if one exchange coupling is stronger than the other, as sketched in the right panel of Figure S11. We note that, as well, there could be a possible exchange pathway between the iron centers forming the double layer (denoted as J_{inter} in Figure S12). Evidently, the Curély model does not take into account this interaction, although it is expected to be smaller than J and J_0 since the distance between the iron centers in the out-of-plane direction (6.178 Å – 6.184 Å) is slightly larger than in the in-plane one (5.986 Å – 6.089 Å).

Following the Curély expression, the susceptibility for a compensated lattice takes the form:

$$\chi = \frac{\beta}{6}\frac{(G^2 + G'^2)W_1 + 2GG'W_2}{(1-u^2v^2)(1-v^2)}$$

where G is the associated Landé factor (in $\mu_B/\hbar$ units), $\beta=1/k_BT$ and with

$$W_1 = (1 + uv)^2(1 + v^2)$$

$$W_2 = 2v(1 + uv)^2 + u(1 - v^2)^2$$

u and v are given by the well-known Langevin function $\left(\mathcal{L}(x) = \coth(x) - \frac{1}{x}\right)$:

$$u = \mathcal{L}(-\beta J_0)$$

$$v = \mathcal{L}(-\beta J)$$

In the present case, we consider $J = J_0$ due to the similar Fe-Fe distances (comprised in between 6.0 and 6.2 Å). This magnetic model has shown that these two J's are strongly correlated (Figure S22-Figure S25) in such a way that the magnetic susceptibility is expected to be insensitive to a large extent to the ratio between these two parameters and many set of parameters reproduce in a close way the experiment. As indicated in the main text, there is no model available for the double-layer structure presented by MUV8. Still, we have seen that a single layer model with 2J's, closely reproduce the experimental observation (in a similar way that a single chain model with J-alternation can reproduce the behaviour of a ladder chain). The addition of a third J parameter, accounting for the inter-plane interaction, would lead to over-parametrization and will not improve the current fit.

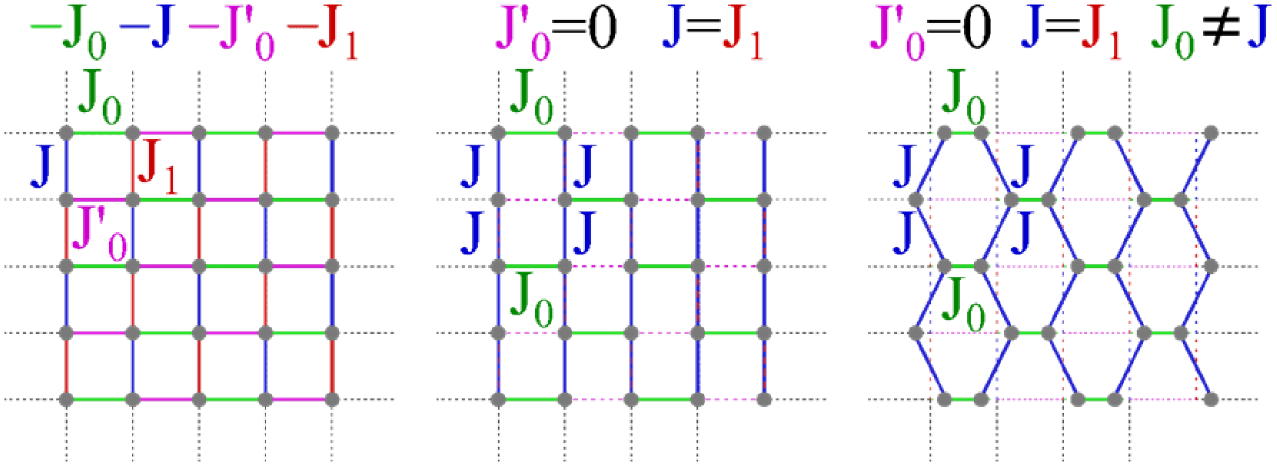

Figure S.11.Example of a transformation from a 2D lattice composed of classical spins and characterized by a square unit cell (left panel) to a hexagonal honeycomb lattice (middle and right panels).

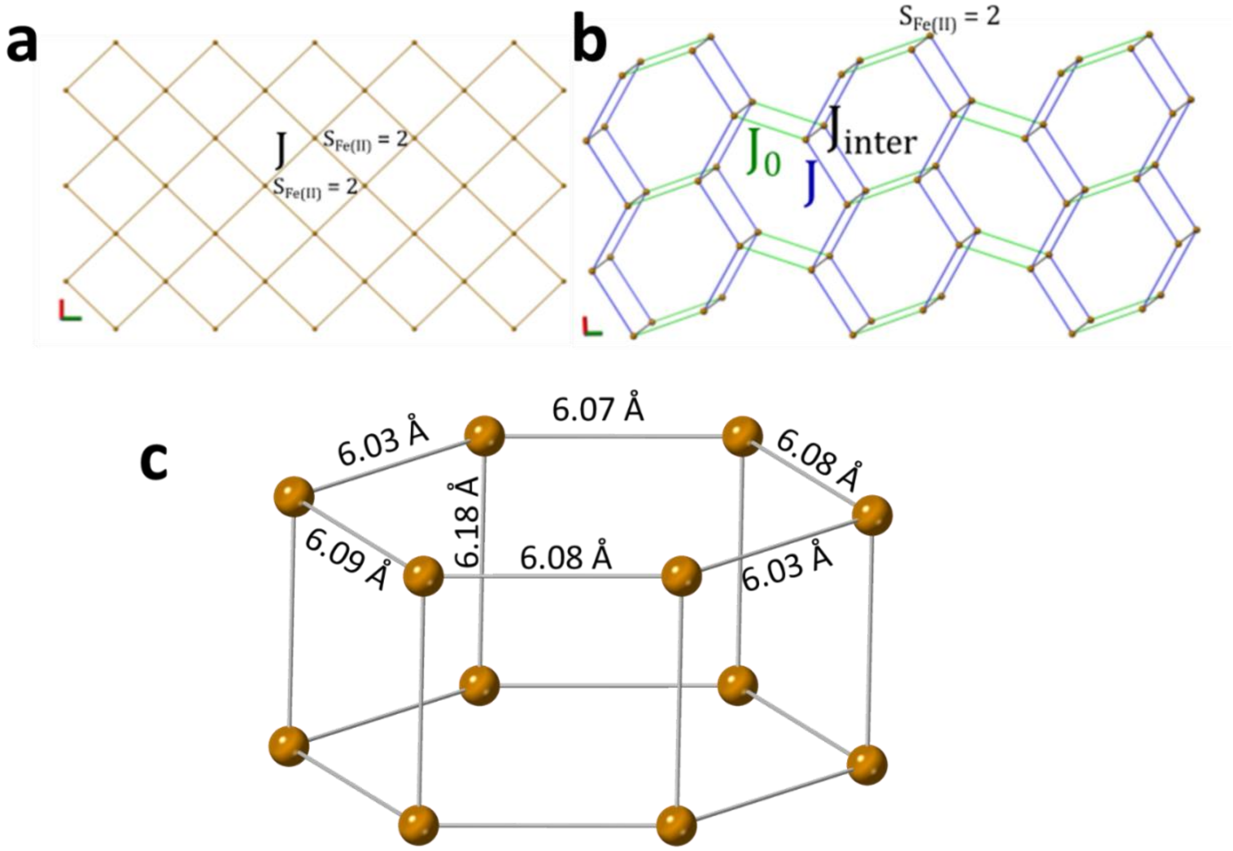

Figure S.12. Magnetic network for **MUV-1** (a) and **MUV-8** (b) compounds. Red, green and blue axis represent the a, b and c axis, respectively. c)Fe-Fe distances in the distorted hexagonal lattice.

Following the Curély formula (considering $H = \sum_{i,j} JS_i \cdot S_j$),[6] the susceptibility for a compensated lattice takes the form:

$$\chi = -\frac{\beta}{6}\frac{(G^2 + G'^2)W_1 + 2GG'W_2}{(1 - u^2v^2)(1 - v^2)}$$

where G is associated Landé factor (in $\mu_B/\hbar$ units), β=1/kT and with

$$W_1 = (1 + uv)^2(1 + v^2)$$

$$W_2 = 2v(1 + uv)^2 + u(1 - v^2)^2$$

u and v are given by the well-known Langevin function:

$$u = \mathcal{L}(-\beta J_0)$$

$$v = \mathcal{L}(-\beta J)$$

The obtained values for J and g are summarized in Table S.2, considering $J = J_0$.

In all compounds, the exchange coupling is antiferromagnetic as denoted by $J < 0$. In the case of the spin-canted systems, the J values are in consonance with the previous reported values for **MUV-1 (Fe)**. In the case of **MUV-1(Co)**, the exchange constant values are lower and, in accordance, they exhibit a lower Néel temperature. **Regarding MUV-1(Mn)**, where no spin-canting is observed, the J values are lower. Finally, **MUV-8(Fe)** exhibits similar Néel temperatures as **MUV-1(Fe)** thus, with similar values of J.

Table S.2.- Summary of the magnetic properties for the different compounds. J and g values for **MUV-1** and **MUV-8** are obtained according to the Lines and Curély expressions, respectively.[5,6]

Structure type	T_N (K)	J (cm^{-1})	g
MUV-1-Cl(Fe)<br>From ref. [7]	20.7	- 22.9 ± 0.4	2.000 ± 0.015
MUV-1-H(Fe)<br>From ref. [7]	20.0	- 23.5 ± 0.2	1.977 ± 0.011
MUV-1-Cl(Co)	11.6	- 20.2 ± 0.4	2.3 ± 0.2
MUV-1-H(Co)	12.4	- 20.8 ± 0.4	2.19 ± 0.15
MUV-1-Cl(Mn)	14.3	- 10.7 ± 0.2	2.2 ± 0.2
MUV-1-H(Mn)	14.8	- 10.41 ± 0.06	2.02 ± 0.09
MUV-8-Cl(Fe)	23.2	- 19.1 ± 0.3	1.97 ± 0.18
MUV-8-CH_3(Fe)	23.4	-25.2 ± 0.5	2.1 ± 0.2

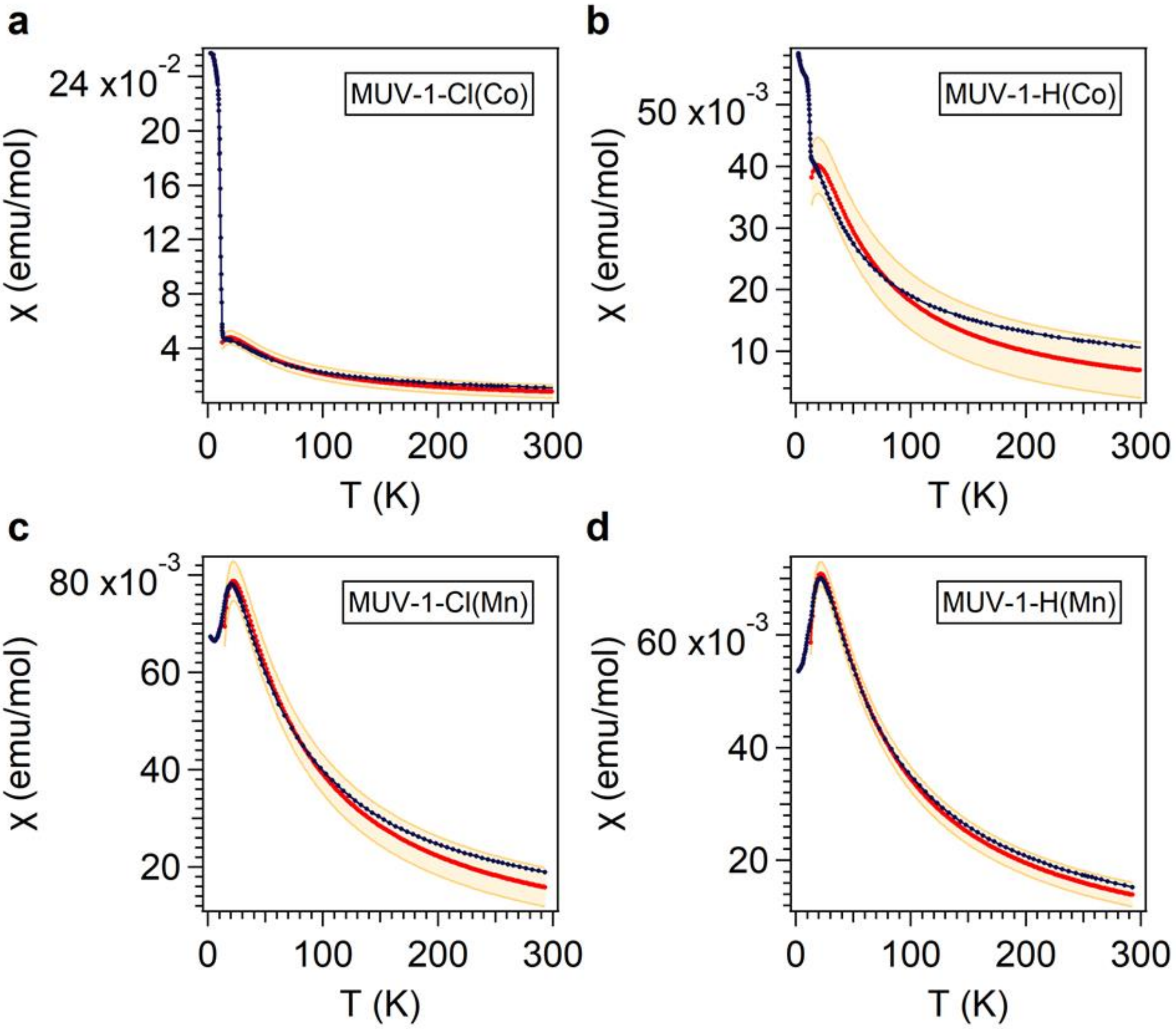

Figure S13. Determination of TN for **MUV-1(Mn)** following the Fisher criteria.[4]

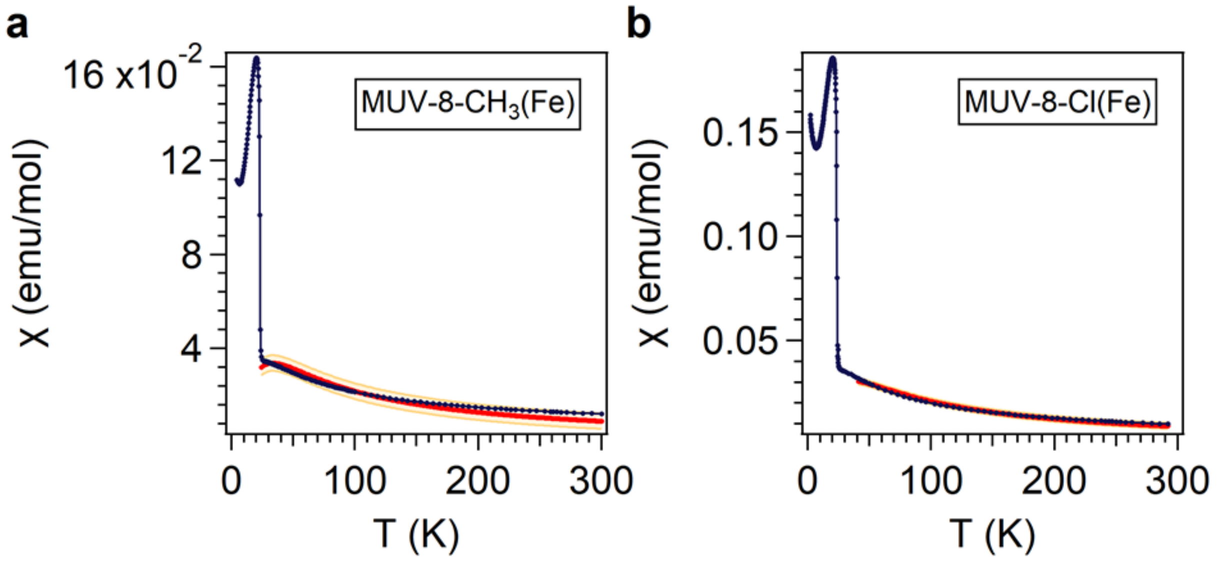

Figure S14. Thermal dependence of χ_m in the temperature range 2-300 K**.** The data have been fitted following the Curély analysis (solid red line).[6] It is represented in yellowish the prediction bands with a confidence interval of 95%..

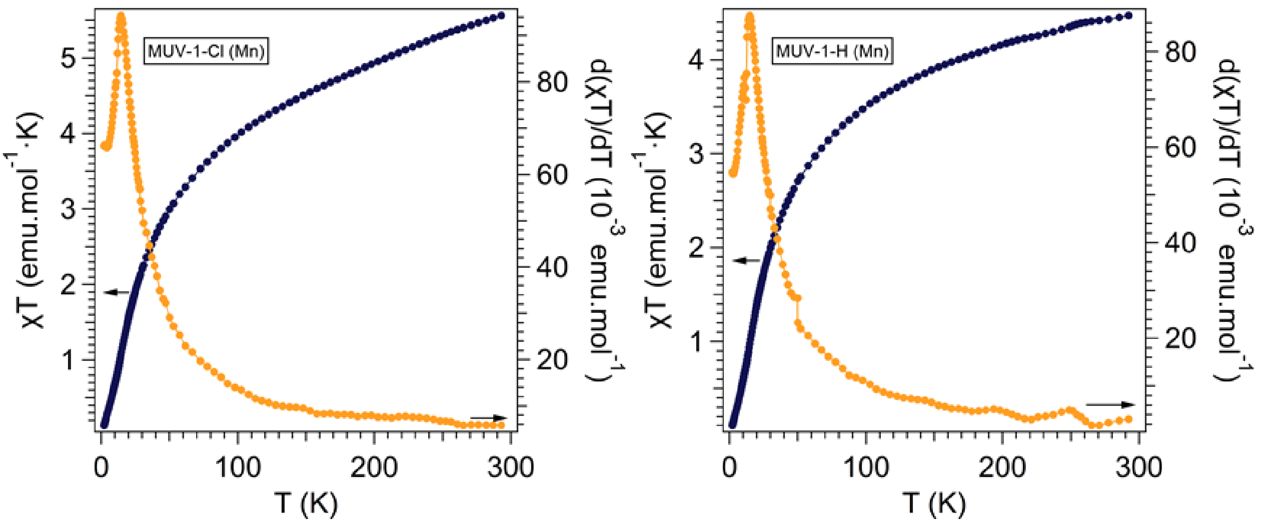

Figure S15. Determination of T_N for **MUV-1(Mn)** following the Fisher criteria.[4]

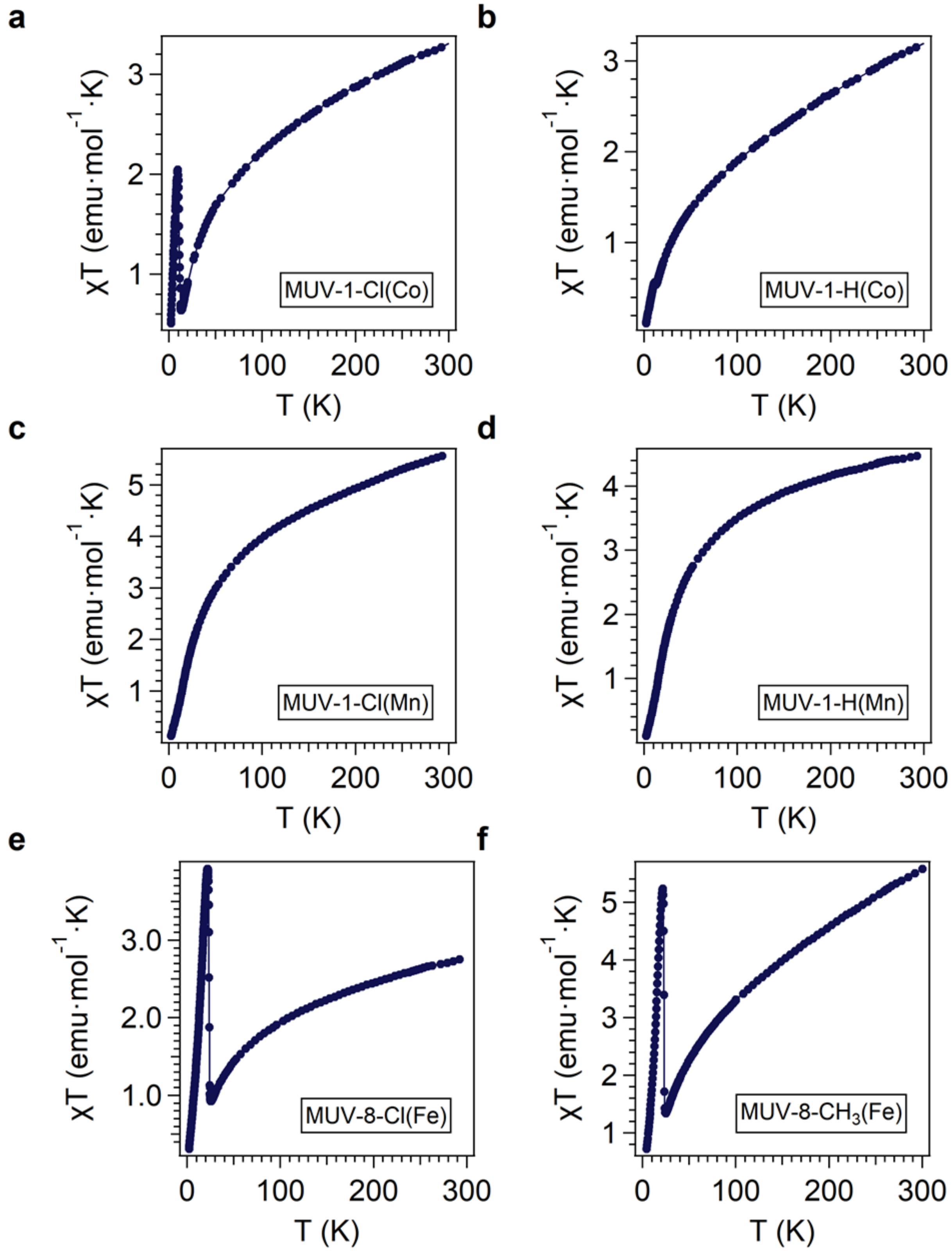

Figure S16. Thermal dependence of the product $\chi_m T$ in the temperature range 2-300 K.

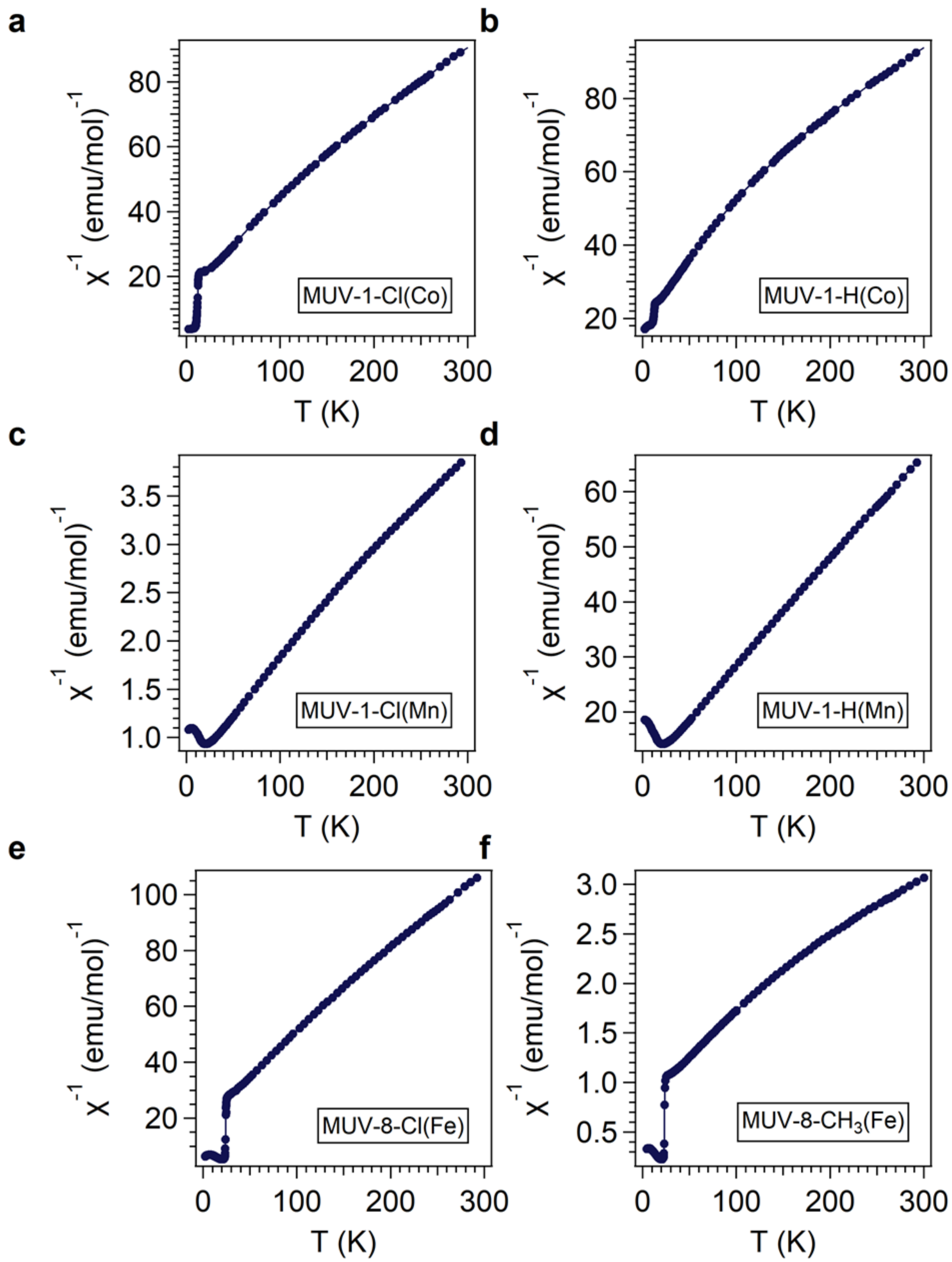

Figure S17. Thermal dependence of χ_m^{-1} in the temperature range 2-300 K.

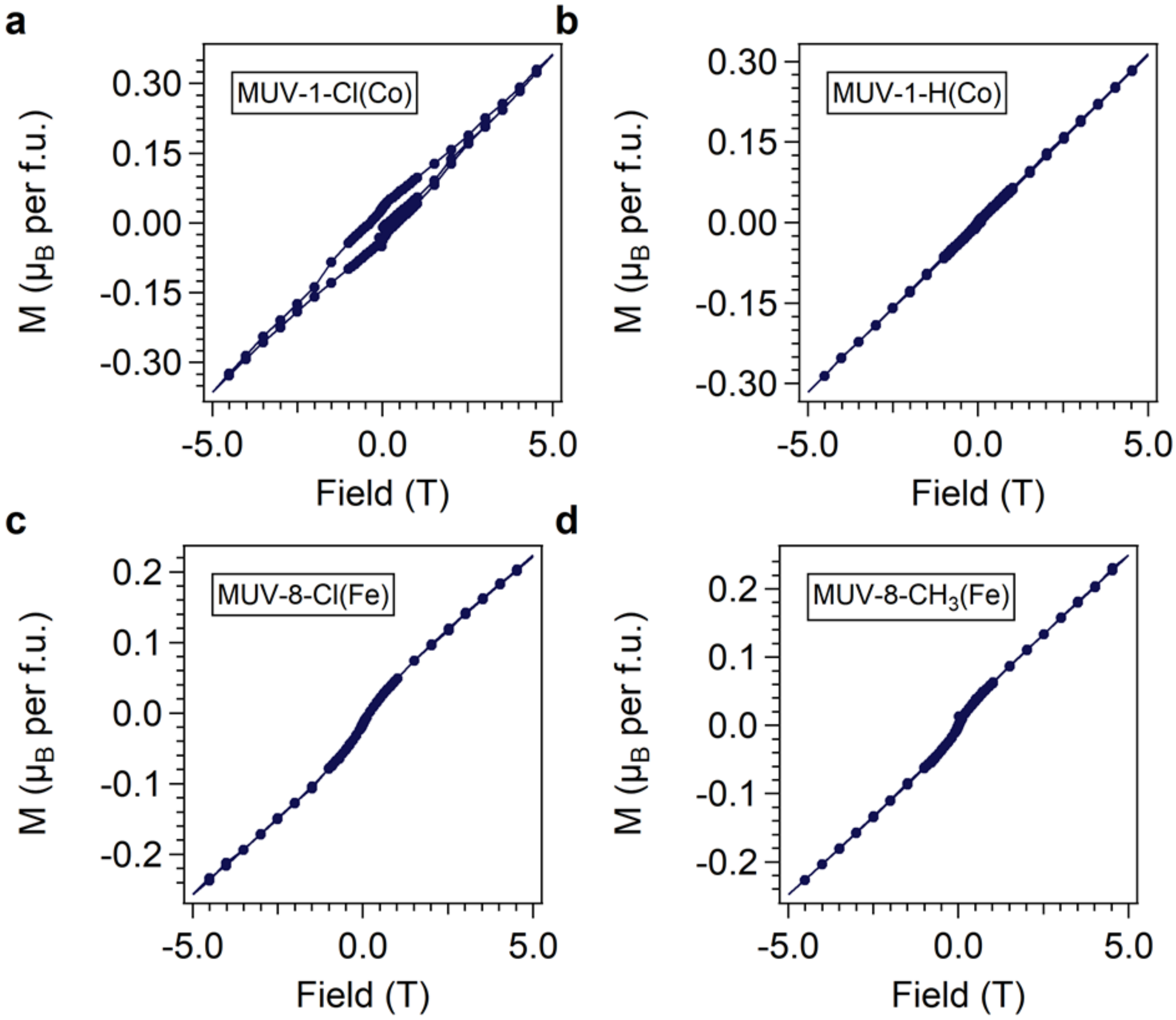

Figure S18. Magnetizations at 2 K.

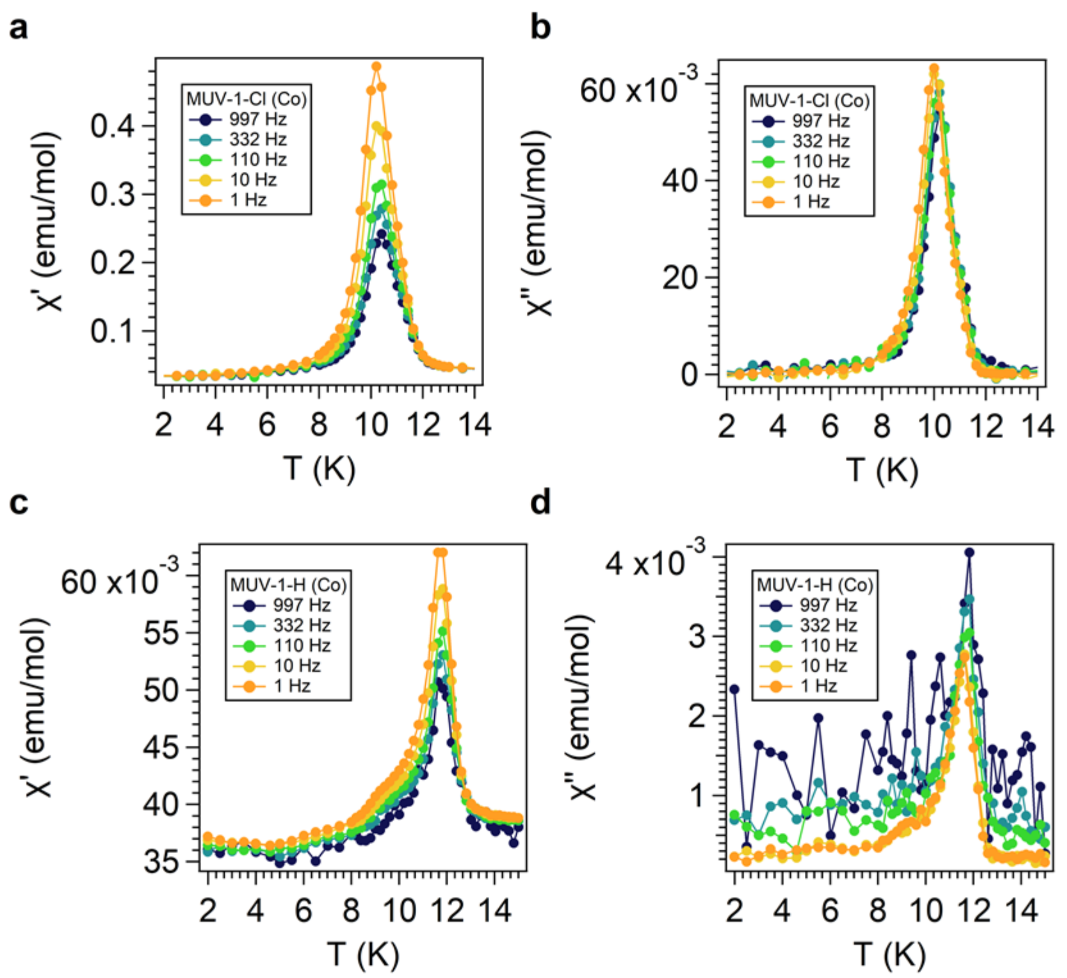

Figure S19. In-phase and out-of-phase dynamic susceptibility of **MUV-1(Co)** measured at different frequencies.

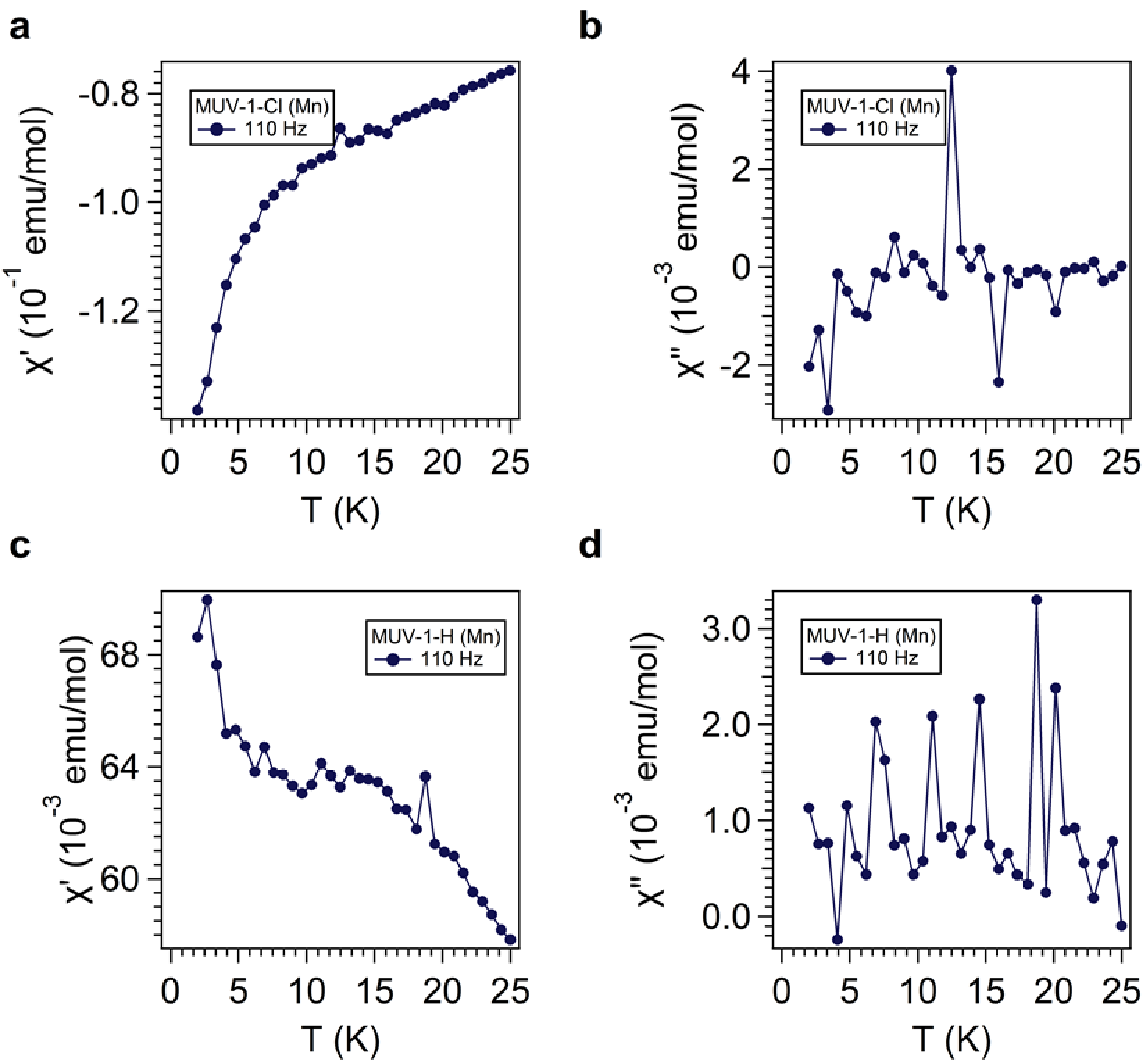

Figure S20. In-phase and out-of-phase dynamic susceptibility of **MUV-1(Mn)**.

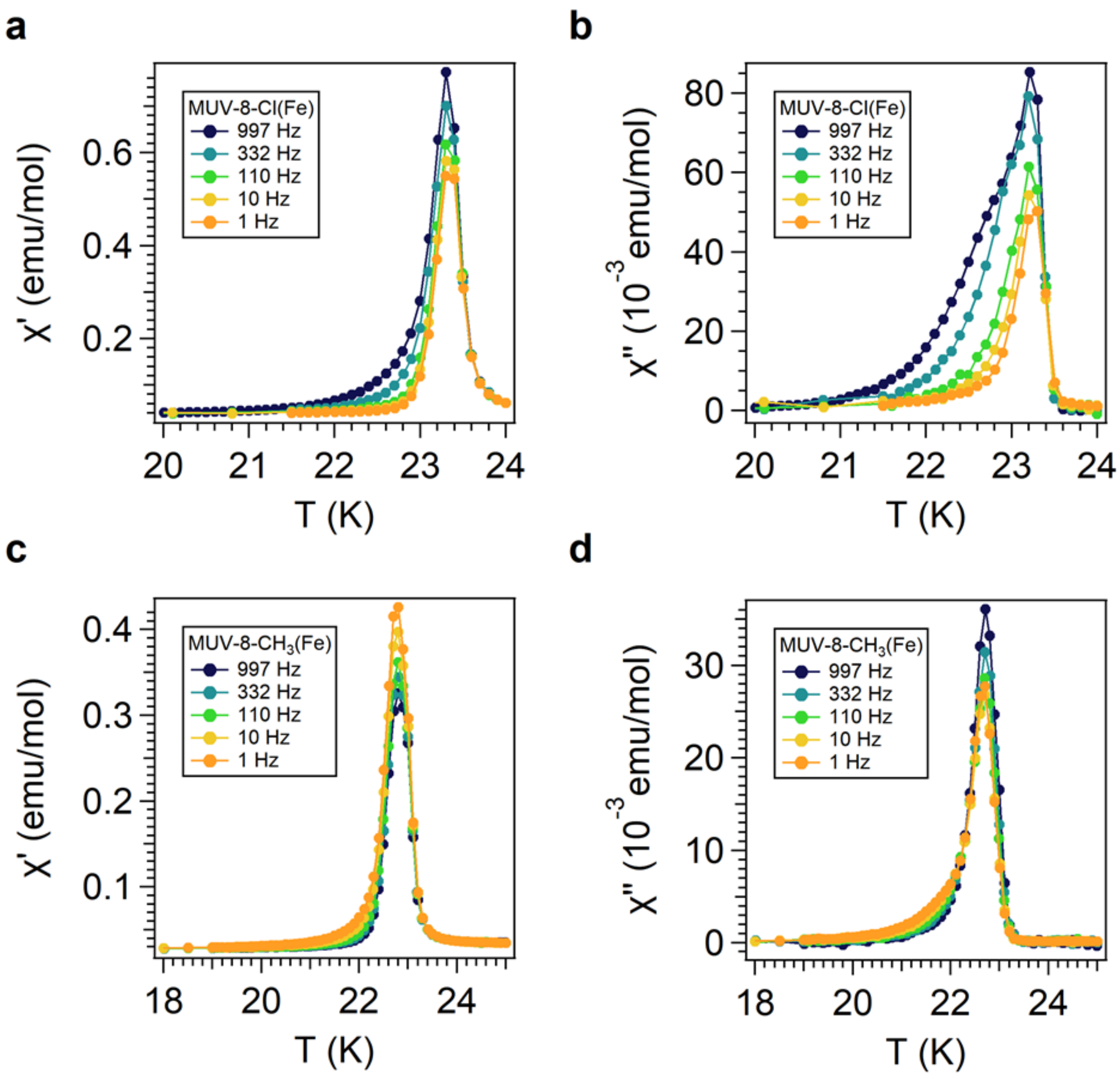

Figure S21. In-phase and out-of-phase dynamic susceptibility of **MUV-8** measured at different frequencies.

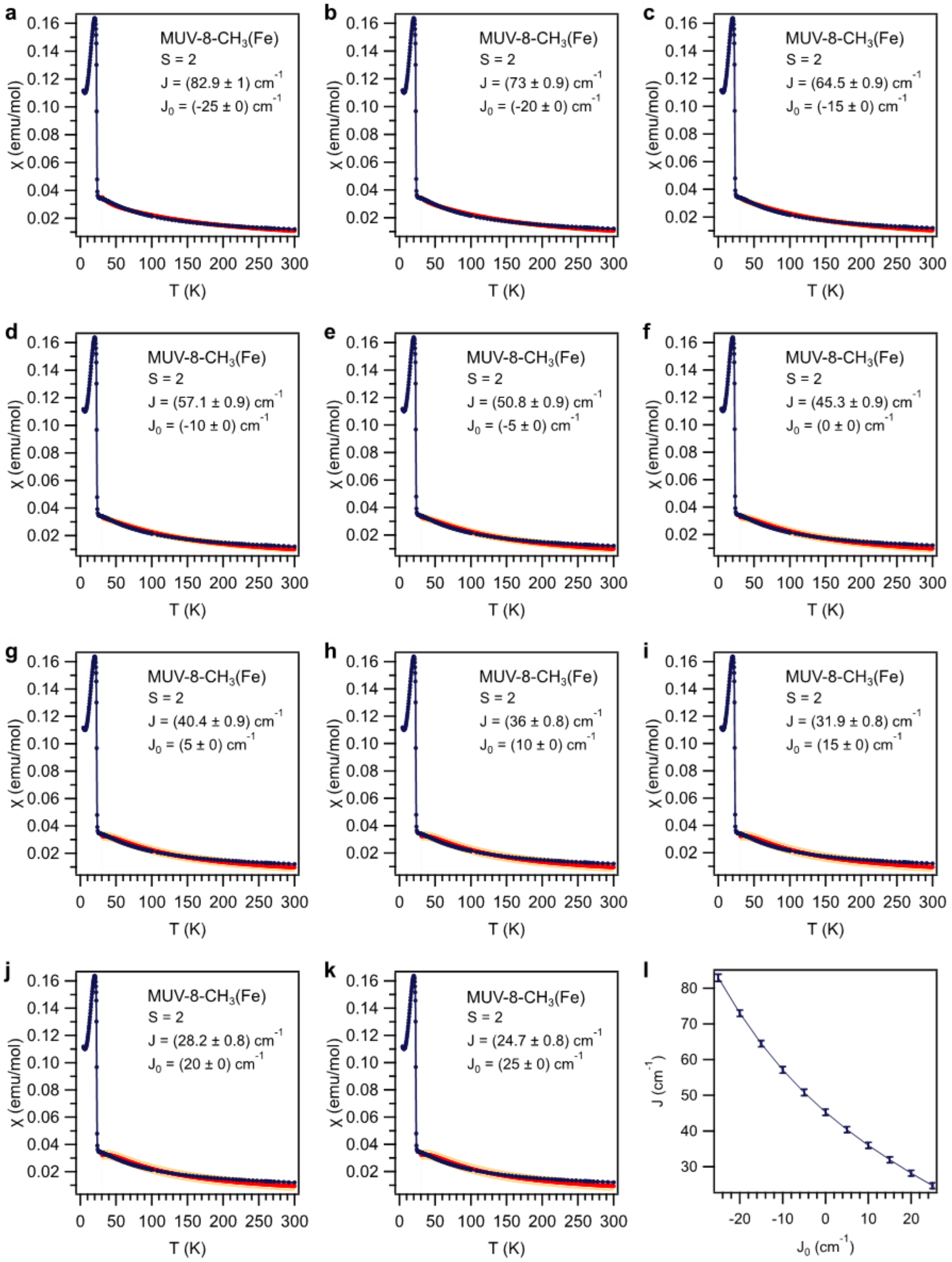

Figure S22. J vs J_0 phase diagram for **MUV-8-CH_3(Fe)**. The data have been fitted following the Curély analysis (solid red line).[6] It is represented in yellowish the prediction bands with a confidence interval of 95%..

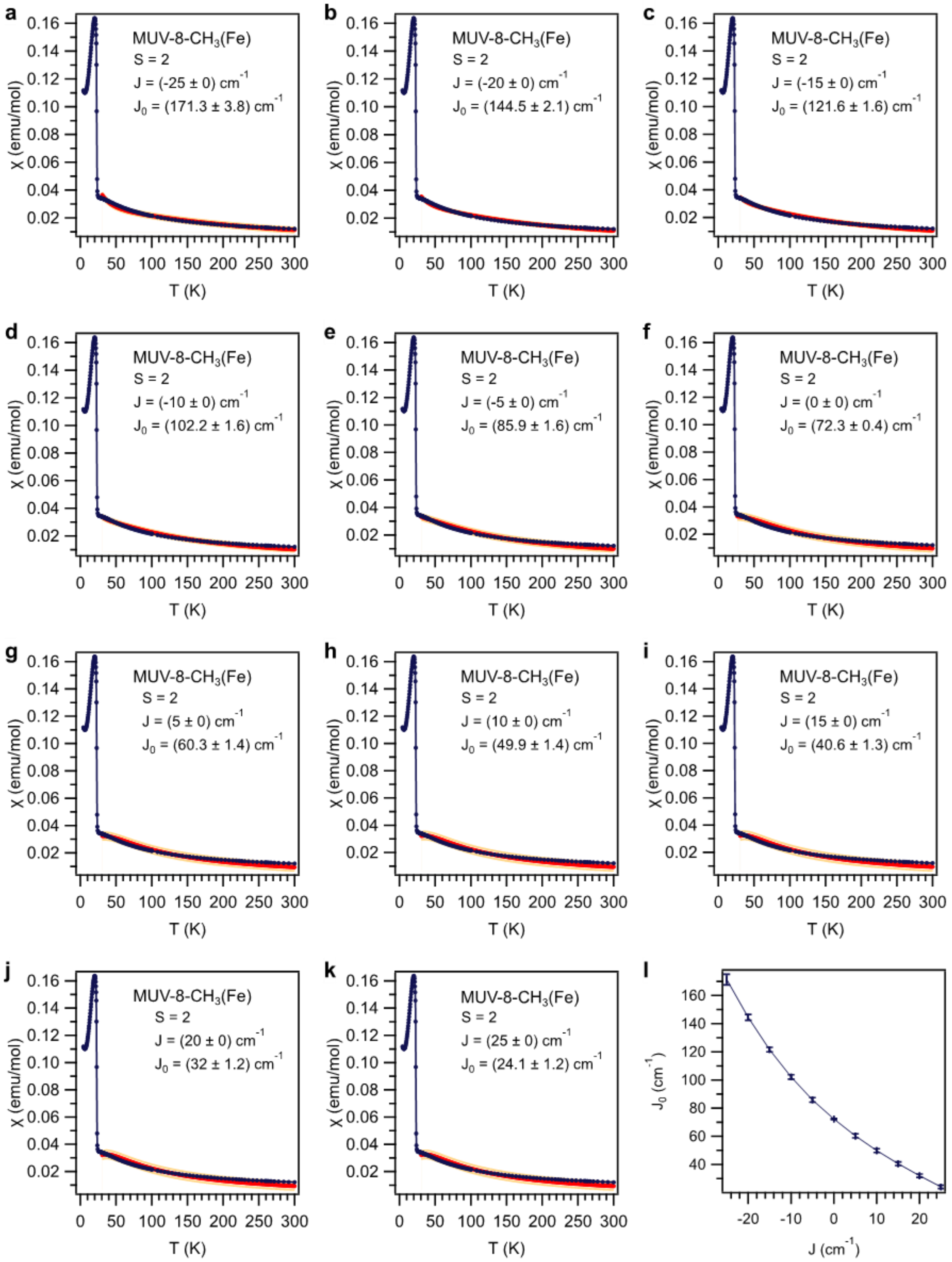

Figure S23. J_0 vs J phase diagram for **MUV-8-CH$_3$(Fe)**. The data have been fitted following the Curély analysis (solid red line).[6] It is represented in yellowish the prediction bands with a confidence interval of 95%..

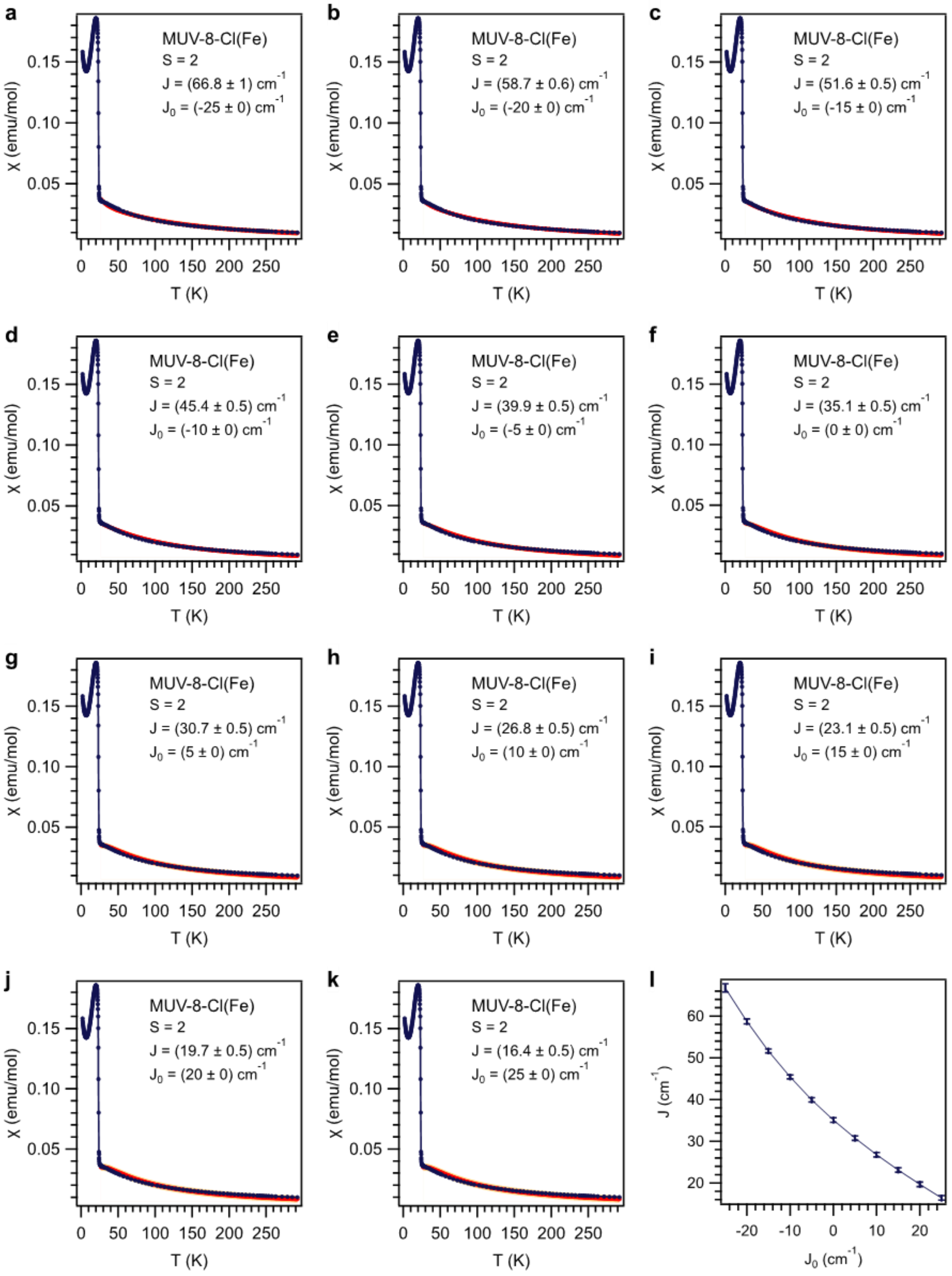

Figure S24. J vs J_0 phase diagram for **MUV-8-Cl(Fe)**. The data have been fitted following the Curély analysis (solid red line).[6] It is represented in yellowish the prediction bands with a confidence interval of 95%..

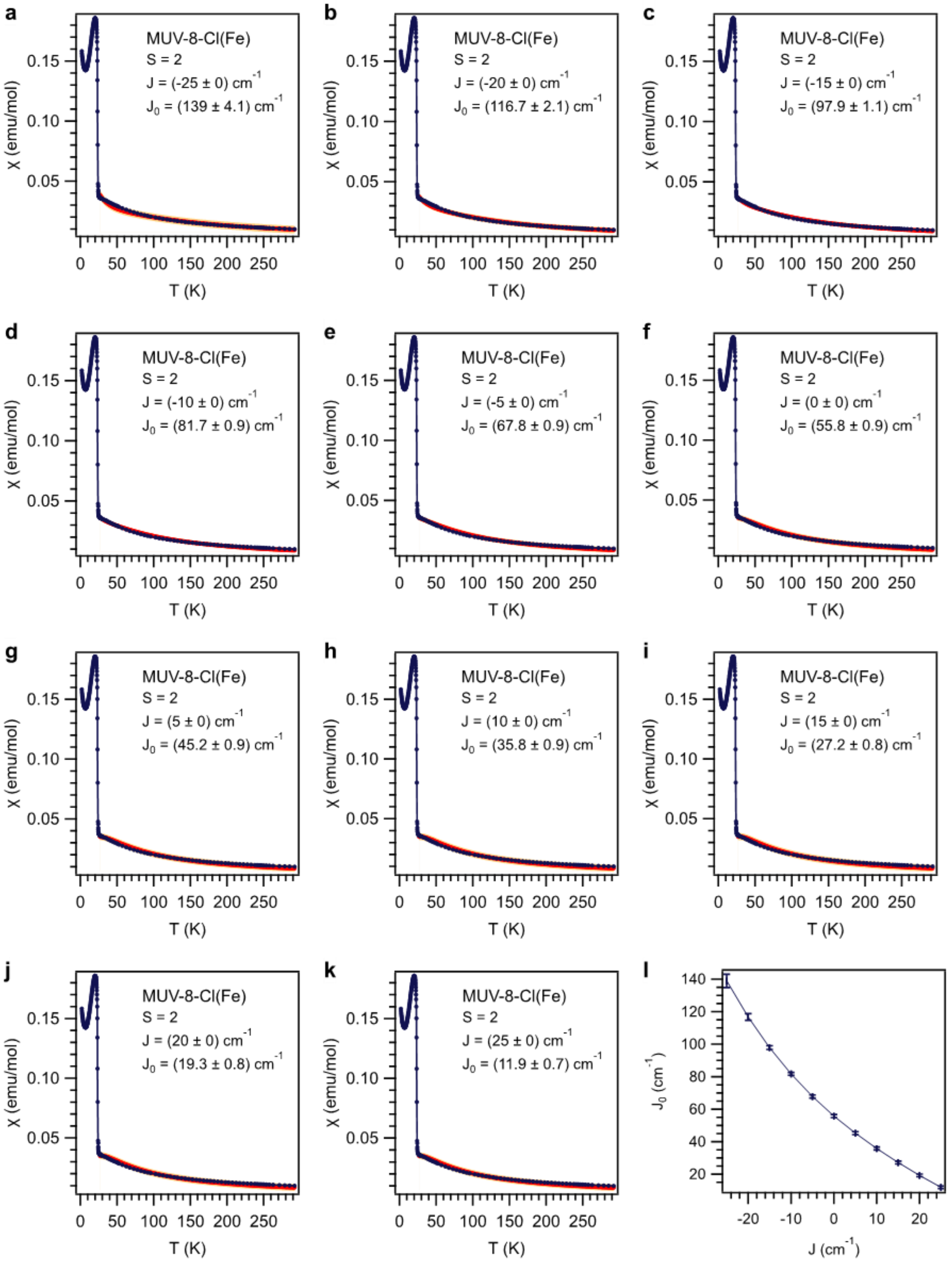

Figure S25. J_0 vs J phase diagram for **MUV-8-Cl(Fe)**. The data have been fitted following the Curély analysis (solid red line).[6] It is represented in yellowish the prediction bands with a confidence interval of 95%..

S4.2 Electron Paramagnetic Resonance measurements

Figure S26 and figure S27 show the EPR spectra of **MUV-1-Cl(Mn)** and **MUV-1-Cl(Co)** for bulk materials at different temperatures. Figure S26 shows a clearly different signal near the ordering temperature (14.3 K). Below this temperature the signal disappears.

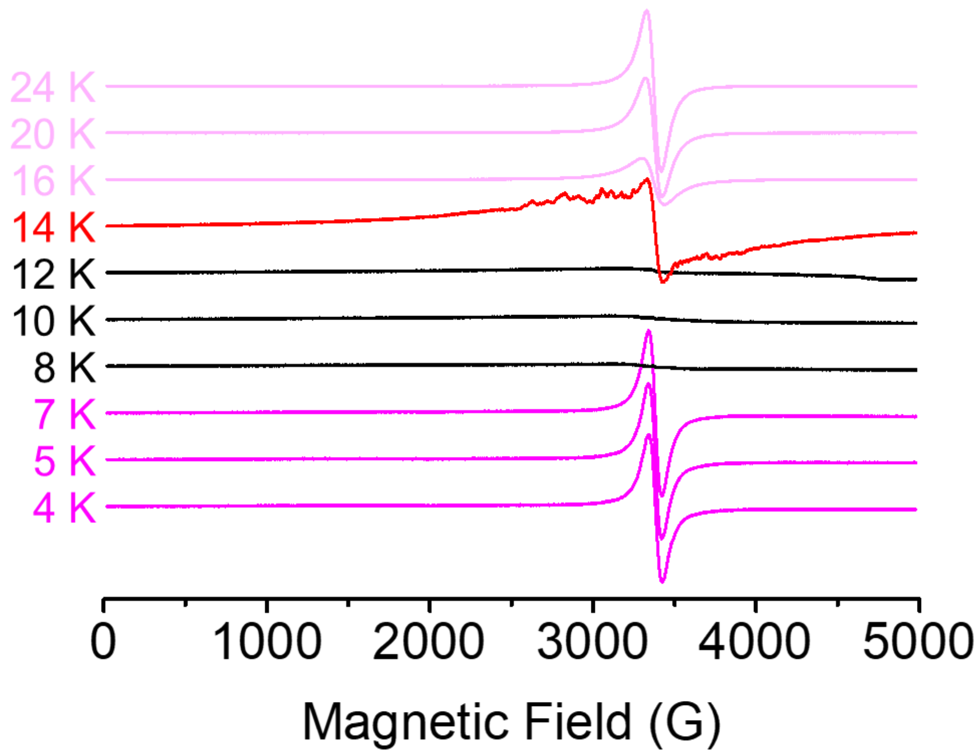

Figure S26. EPR spectra of **MUV-1-Cl(Mn)** at different temperatures.

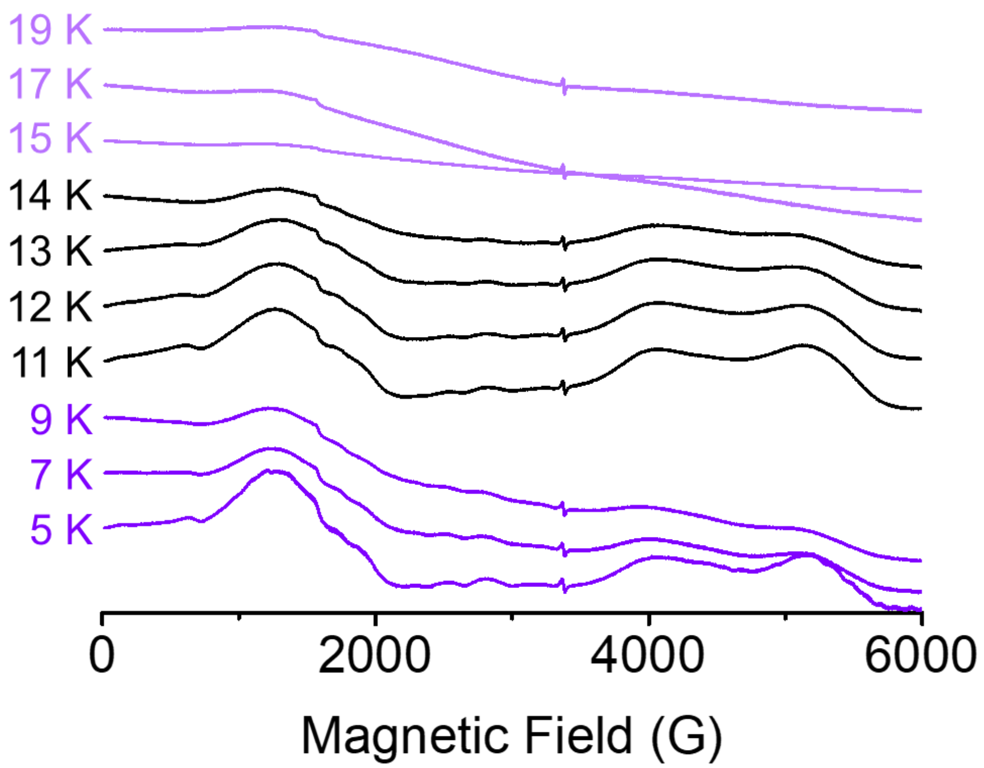

Figure S27. EPR spectra **MUV-1-Cl(Co)** at different temperatures.

S5. Two-dimensional approach and characterization

MUV-1-Cl(Co), MUV-1-H(Co), **MUV-8-Cl(Fe)** and **MUV-8-CH_3(Fe)** were mechanically exfoliated by mechanical methods (as commonly known as the *Scotch-tape* method, developed for obtaining graphene from graphite), using an adhesive plastic film of 80 µm thick tape from Ultron Systems. The obtained flakes were deposited onto silicon substrates with 285 nm of thermally grown SiO_2 and inspected by different microscopies such us optical microscopy and atomic force microscopy.

S5.1 Atomic Force Microscopy of the few layers of MUV-1-Cl(Co), MUV-1-H(Co), MUV-8-Cl(Fe) and MUV-8-CH_3(Fe)

In this section, it is presented a more detail characterization of the few layers shown in the main manuscript. The thickness of the flakes was determined by atomic force microscopy (Nanoscope IVa Multimode Scanning Probe Microscope, Bruker) in tapping mode.

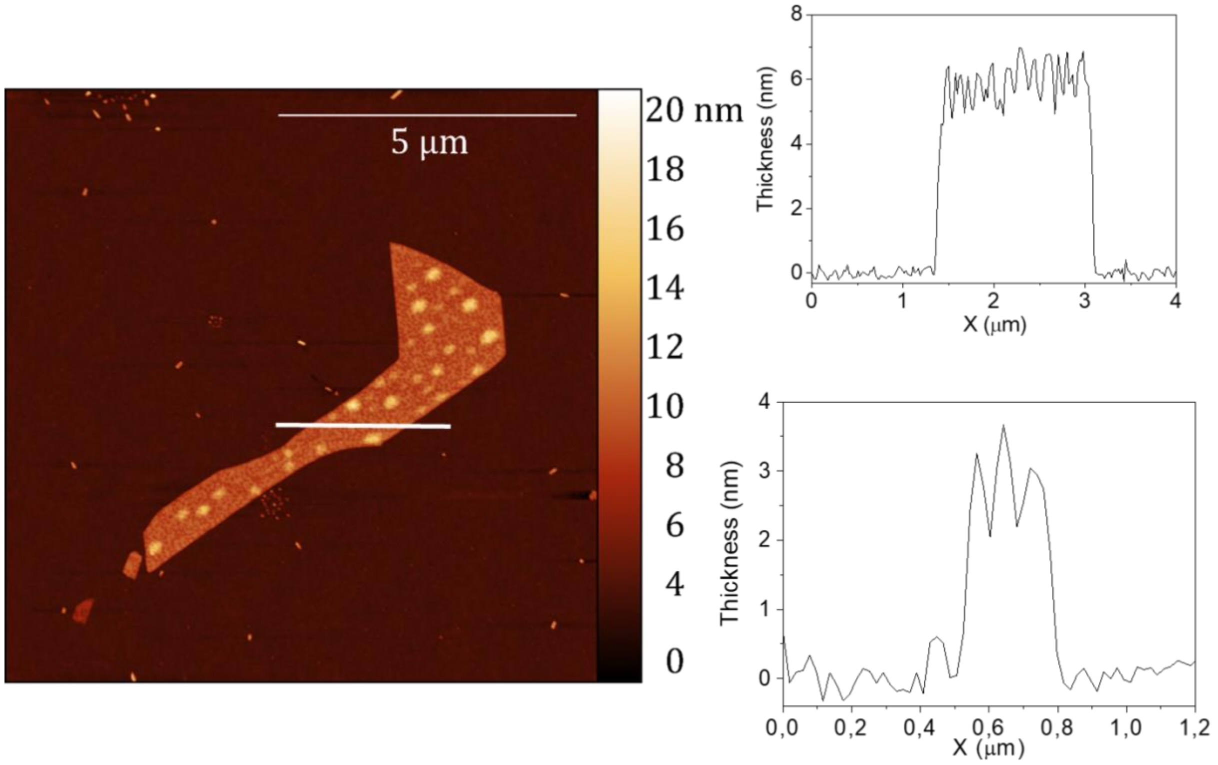

Figure S28. Atomic Force Microscopy images of **MUV-1-Cl(Co)** flakes with its corresponding height profiles.

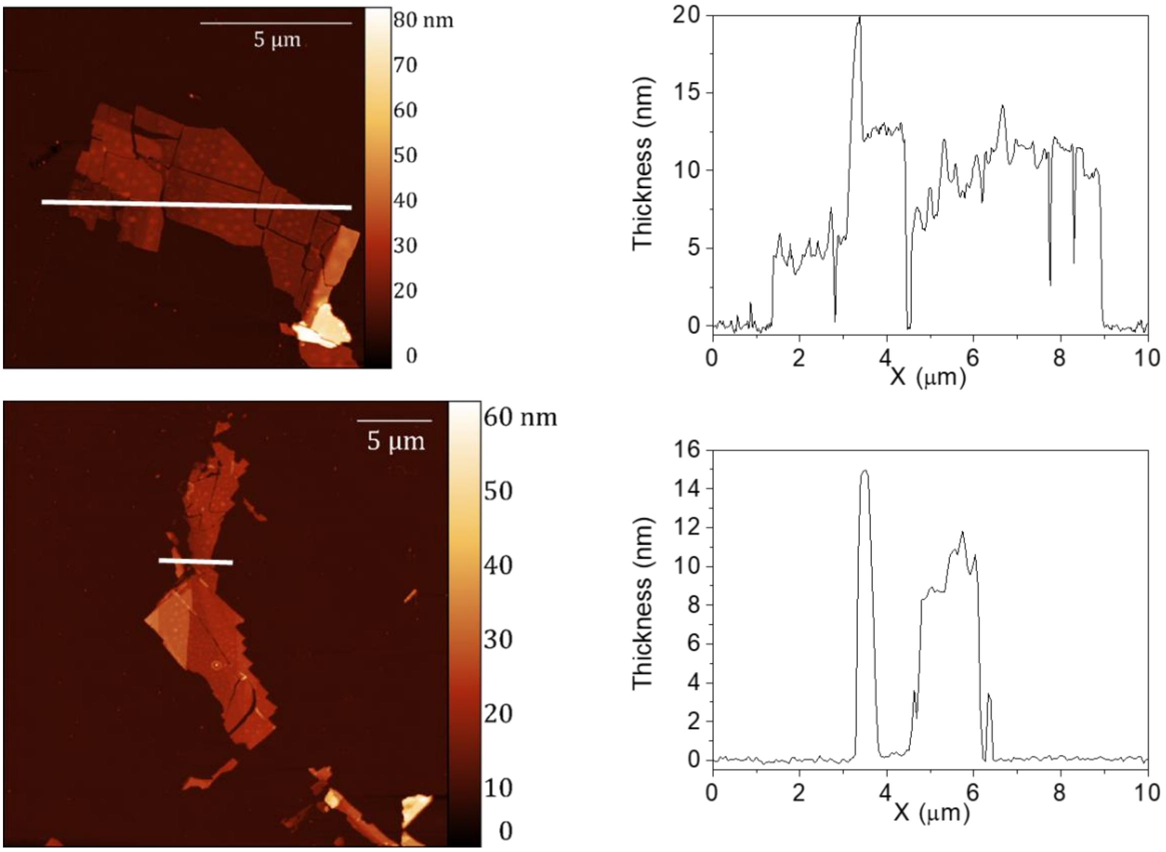

Figure S29. Atomic Force Microscopy images of **MUV-1-H(Co)** flakes with its corresponding height profiles.

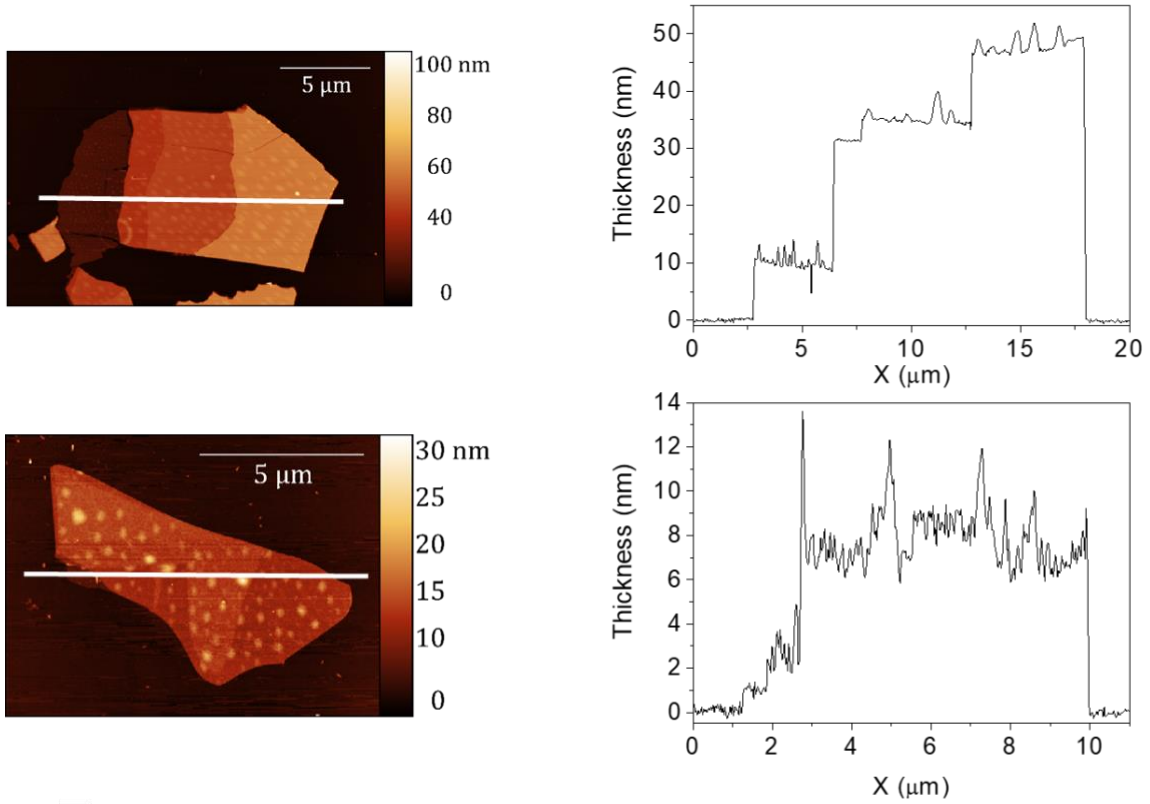

Figure S30. Atomic Force Microscopy images of **MUV-8-Cl(Fe)** flakes with its corresponding height profiles.

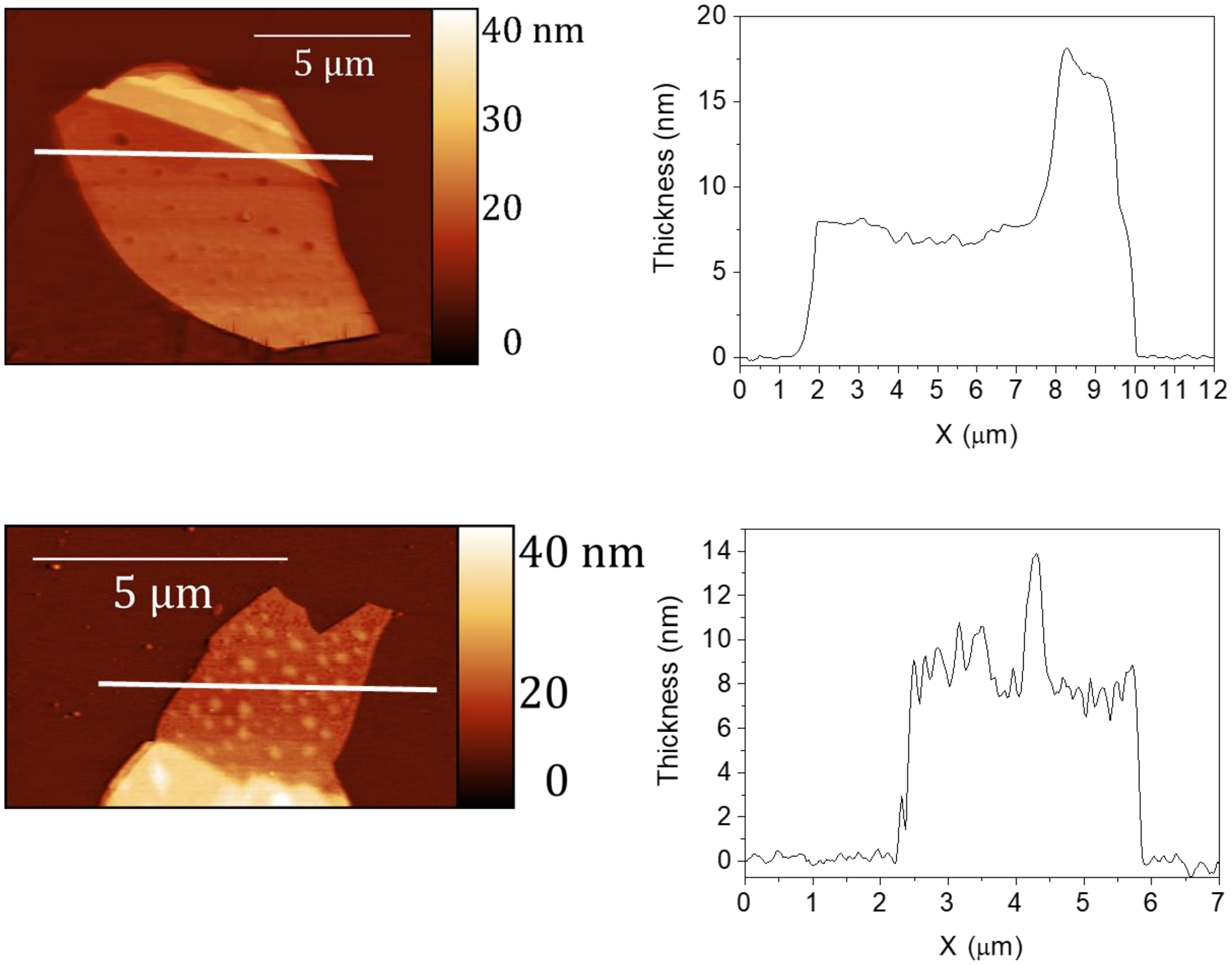

Figure S31. Atomic Force Microscopy images of **MUV-8-CH$_3$(Fe)** flakes with its corresponding height profiles.

S5.2. Raman spectroscopy in bulk and two-dimensional layers of MUV-1-X(Co) and MUV-8-X(Fe).

Raman spectra were acquired with a micro-Raman (model XploRA ONE from Horiba, Kyoto, Japan) with a grating of 2400 gr/mm, slit of 50 µm, and hole of 500 µm. The employed wavelengths for the bulk crystals were 532 nm, 638 nm, and 785 nm and 532 nm for the thin-layers. The power density of the laser used for spectra measured at 532 nm was 1.0 mW/μm^2, for spectra measured at 638 nm it was and 2.5 mW/μm^2 and for those spectra measured at 785 nm it was 3.3 mW/μm^2.

The Raman spectrum recorded for the flakes deposited onto Si/285 nm SiO_2 substrates were performed employing the wavelength of 532 nm.

In addition, in order to assess the nature of the mechanically exfoliated thin-layers, it was measured the Raman spectra of flakes with different thicknesses on Si/285 nm SiO_2 substrates (Figures S3-S34). The thickness of the flakes where the Raman spectra were measured was determined by atomic force microscopy (Nanoscope IVa Multimode Scanning Probe Microscope, Bruker) in tapping mode (Figures S31-S33).

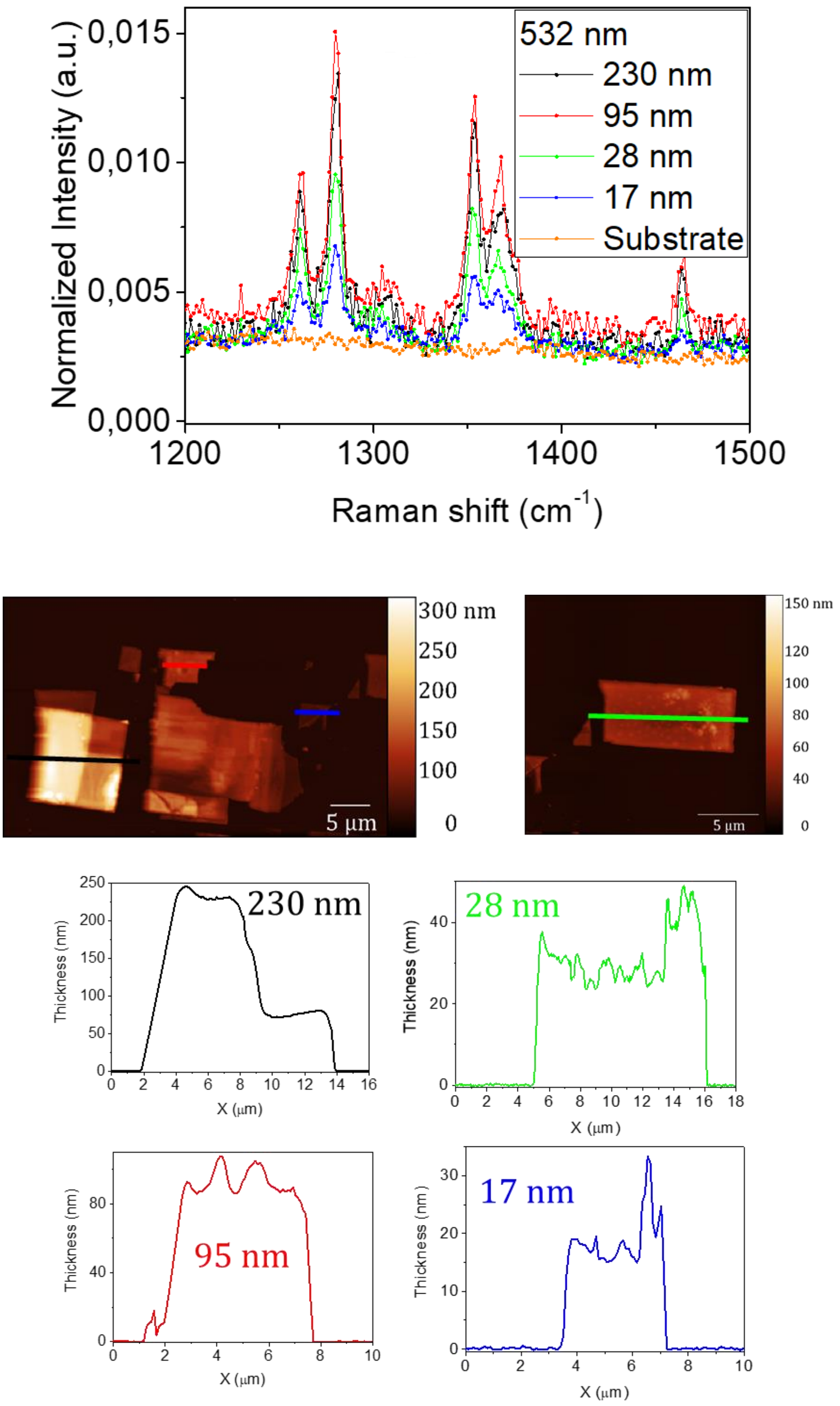

Figure S32. Raman spectra and the Atomic Force Microscopy images of **MUV-1-H(Co)** flakes with its corresponding height profiles.

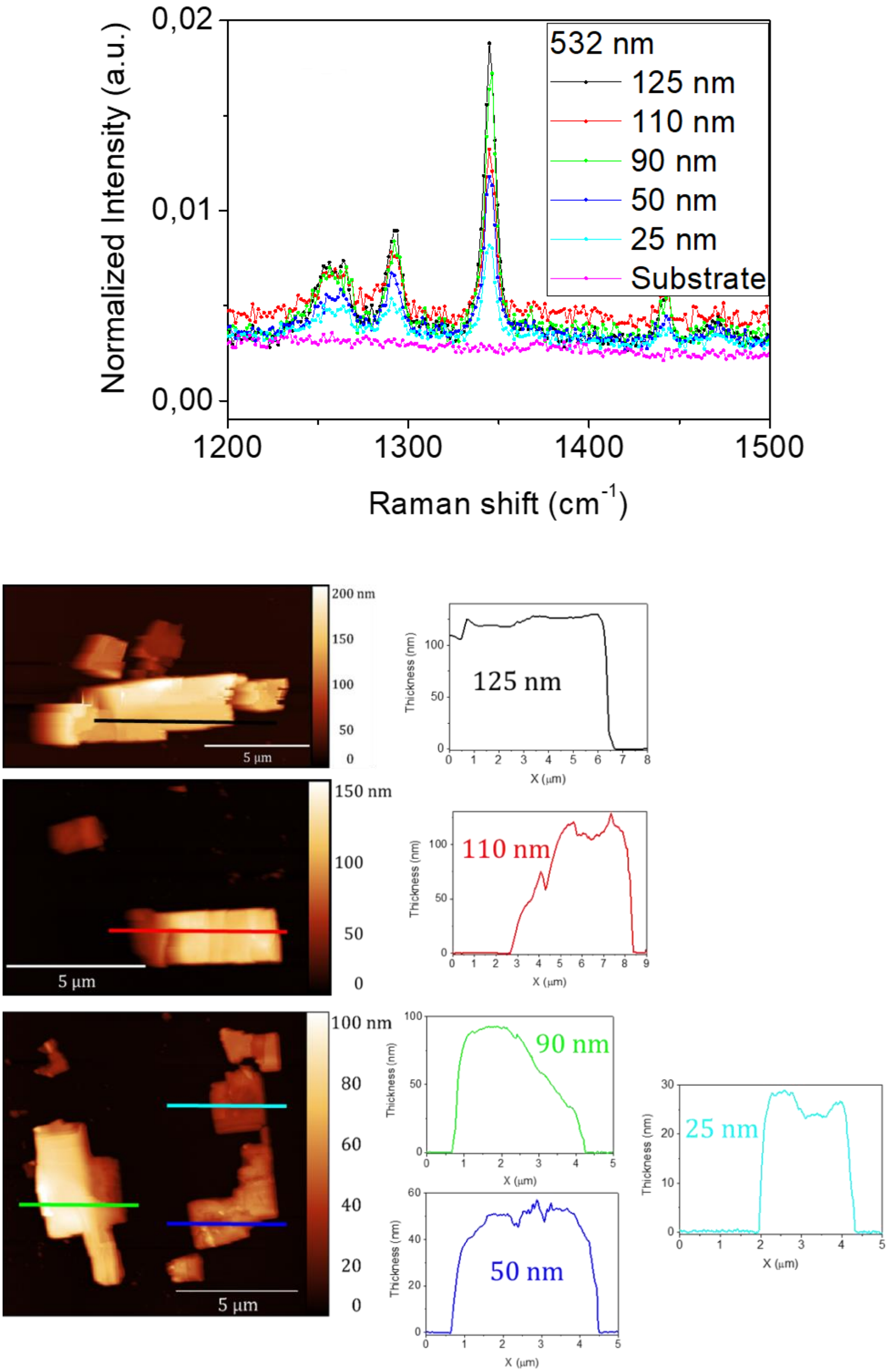

Figure S33. Raman spectra and the Atomic Force Microscopy images of **MUV-1-Cl(Co)** flakes with its corresponding height profiles.

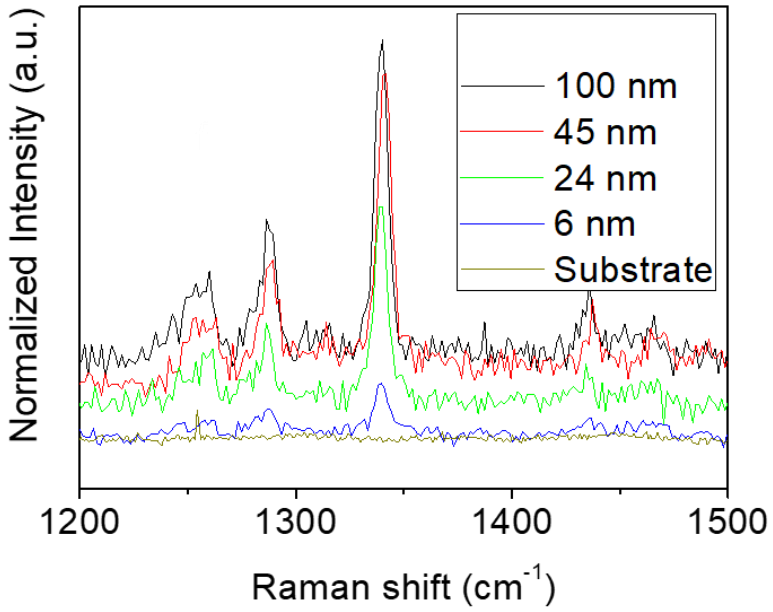
Normalized Intensity (a.u.)
100 nm
45 nm
24 nm
6 nm
Substrate
1200
1300
1400
1500
Raman shift (cm-1)

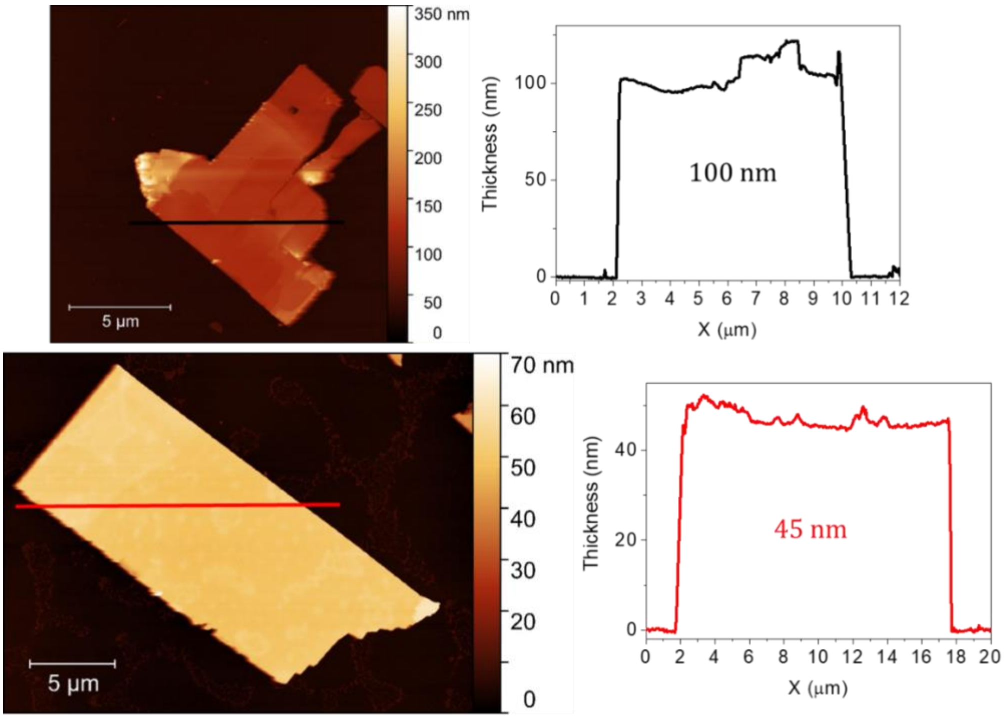
350 nm
300
250
200
150
100
50
0
5 µm
Thickness (nm)
100
50
0
0 1 2 3 4 5 6 7 8 9 10 11 12
X (µm)
100 nm
70 nm
60
50
40
30
20
0
5 µm
Thickness (nm)
40
20
0
0 2 4 6 8 10 12 14 16 18 20
X (µm)
45 nm

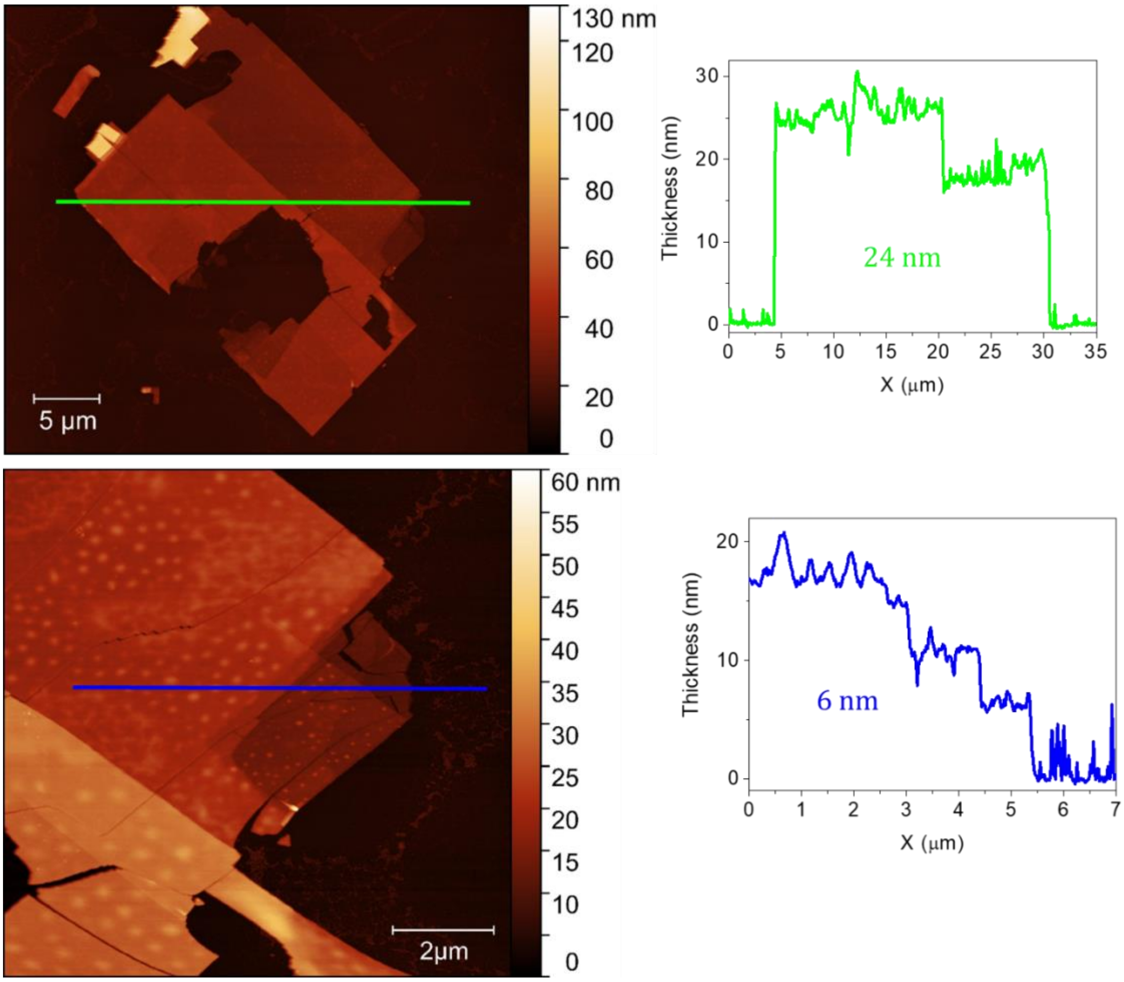

Figure S34. Raman spectra and the Atomic Force Microscopy images of **MUV-8-Cl(Fe)** flakes with its corresponding height profiles.

S6. Bibliography

(1) Sheldrick, G. M. Crystal Structure Refinement with SHELXL. *Acta Crystallogr. Sect. C Struct. Chem.* **2015**, *71* (1), 3–8.

(2) Dolomanov, O. V.; Bourhis, L. J.; Gildea, R. J.; Howard, J. A. K.; Puschmann, H. OLEX2 : A Complete Structure Solution, Refinement and Analysis Program. *J. Appl. Crystallogr.* **2009**, *42* (2), 339–341.

(3) Sechovsky, V. Encyclopedia of Materials: Science and Technology : Magnetism in Solids: General Introduction. *Elsevier Sci. Ltd.* **2000**, 1–15.

(4) Fisher, M. E. Relation between the Specific Heat and Susceptibility of an Antiferromagnet. *Philos. Mag.* **1962**, *7* (82), 1731–1743.

(5) Lines, M. E. The Quadratic-Layer Antiferromagnet. *J. Phys. Chem. Solids* **1970**, *31* (1), 101–116.

(6) Curély, J.; Lloret, F.; Julve, M. Thermodynamics of the Two-Dimensional Heisenberg Classical Honeycomb Lattice. *Phys. Rev. B* **1998**, *58* (17), 11465–11483.

(7) López-Cabrelles, J.; Mañas-Valero, S.; Vitórica-Yrezábal, I. J.; Bereciartua, P. J.; Rodríguez-Velamazán, J. A.; Waerenborgh, J. C.; Vieira, B. J. C.; Davidovikj, D.; Steeneken, P. G.; van der Zant, H. S. J.; Mínguez Espallargas, G.; Coronado, E. Isoreticular Two-Dimensional Magnetic Coordination Polymers Prepared through Pre-Synthetic Ligand Functionalization. *Nat. Chem.* **2018**, *10* (10), 1001–1007.
